# Echoes of Evolution in Holocentridae: Harmony Between Phylogeny, Morphology and Acoustics

**DOI:** 10.1002/ece3.74152

**Published:** 2026-08-19

**Authors:** Marine Banse, Maarten Van Steenberge, Gontran Sonet, Denisa Magda, Yves Corbisier de Harbonnier de Cobreville, David Lecchini, Terry J. Donaldson, Eric Parmentier

**Affiliations:** ^1^ Laboratory of Functional and Evolutionary Morphology University of Liège Liège Belgium; ^2^ Operational Directorate Taxonomy and Phylogeny Royal Belgian Institute of Natural Sciences Brussels Belgium; ^3^ Joint Experimental Molecular Unit (JEMU), Operational Directorate Taxonomy and Phylogeny Royal Belgian Institute of Natural Sciences Brussels Belgium; ^4^ Center for Insular Research and Observatory of the Environment (CRIOBE) Moorea French Polynesia; ^5^ University of Guam Marine Laboratory, Guam EPSCoR Mangilao Guam USA

**Keywords:** acoustic communication, diversification, evolution, Holocentridae, sonic apparatus, sound

## Abstract

Holocentridae are nocturnal reef fishes that rely on acoustic communication in various behavioral contexts. Using a multidisciplinary approach combining phylogenetics, morphology, and acoustic data from 53 species, we reconstructed the evolutionary history of this communication system within the family. Our results reveal two main clades corresponding to the subfamilies Myripristinae and Holocentrinae, with congruent differences in both the morphology of the sound‐producing apparatus and acoustic features. Interestingly, at finer taxonomic scales, closely related species often share similar sound‐producing structures but can produce distinct sounds. This suggests that the evolution of acoustic communication in Holocentridae follows a three‐step diversification pattern: (1) the emergence of structures enabling sound production; (2) differentiation of sound‐producing mechanisms within each subfamily, likely constrained by internal factors such as the swim bladder morphology; and (3) further diversification through differentiated use of conserved mechanisms, consistent with a role of neurophysiological modulation. This scenario mirrors patterns observed in other functional traits, where morphological innovations open new functional pathways, followed by fine‐scale diversification based on the flexible exploitation of similar structures. It provides one of the first evolutionary reconstructions of sound production mechanisms in fishes, expanding our understanding of the evolution of complex signaling systems.

## Graphical abstract

Using phylogenetic, morphological, and acoustic data from 53 species, we reconstructed the evolution of acoustic communication in nocturnal reef fishes of the family Holocentridae. Our results reveal two major clades with distinct sound‐producing morphologies and acoustic features, and suggest a three‐step diversification process involving the emergence of sound‐producing structures, subfamily‐level differentiation constrained by morphology, and fine‐scale acoustic diversification via differential use of conserved mechanisms. This provides one of the first evolutionary reconstructions of sound production systems in fishes and sheds light on the evolution of complex signaling traits.

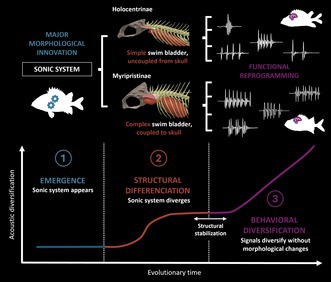

## 1 Introduction

The use of sounds as a means of communication in teleost fish is a behavior whose significance has been exponentially demonstrated over several decades. Today, about a thousand fish species from 133 families and 33 orders are recognized as using voluntary sounds for social communication (Looby et al. 2022), and this number is continuously increasing. In other taxa, such as primates, manatees, bats, birds, amphibians, and insects (Henry 1994; Jones 1997; Borkin et al. 2004; Braune et al. 2008; Cole 2008; Burton and Nietsch 2010; Wilkins et al. 2018; Reyes‐Arias et al. 2023; Spezie et al. 2023; Irwin, Bensch, and Price 2001; Irwin, Irwin, and Price 2001), species‐specific acoustic signals are primarily regarded as prezygotic barriers that contribute, wholly or partially, to preventing interbreeding. In teleosts, the species‐specific nature of sounds has also been statistically supported in different taxa such as in Pomacentridae (Spanier 1979; Parmentier et al. 2009), Serrasalmidae (Mélotte et al. 2016; Raick et al. 2020, 2023), Gobiidae (Horvatić et al. 2021) or Sciaenidae (Picciulin et al. 2021). However, most studies addressing this question have involved limited numbers of species, leaving open the question of how acoustic distinctiveness holds across larger taxonomic samples. A recent study based on the analysis of the sounds of 33 holocentrid species, a sample size significantly larger than in all previous studies, has challenged the notion of strict species‐specificity in teleost sounds (Banse, Bertimes, et al. 2024). However, the ability to distinguish taxa based on acoustic features increases with taxonomic rank, with discrimination becoming more effective at higher hierarchical levels. This pattern suggests that acoustic traits could reflect evolutionary divergence and might be linked to differences in the sound‐producing mechanism itself.

In families such as in Ophidiidae (Casadevall et al. 1996) or Serrasalmidae (Mélotte et al. 2019), significant morphological differences in sound‐producing mechanisms occur at the genus or species level. In other taxa, such as in Pomacentridae (Colleye et al. 2011), Balistidae (Raick et al. 2018) and Batrachoididae (Rice and Bass 2009), the same type of sound‐producing mechanism is shared by all species within the families and may therefore be used as a synapomorphy. These contrasting patterns raise the question of whether and how the evolution of sound‐producing mechanisms is linked to species diversification within a given clade. Despite the growing number of studies describing fish sounds, sonic mechanisms, and acoustic behaviors, the evolutionary role of acoustic communication in shaping species diversification has rarely been investigated in teleosts (Parmentier et al. 2016; Horvatić et al. 2021). Fundamental questions remain: how did sound‐producing mechanisms evolve, and to what extent is acoustic signal diversity linked to morphological diversification? We hypothesized that species recognition may have contributed to the evolution of sound‐producing mechanisms, enabling the production of distinct sounds. Addressing this requires studying a taxonomic group that combines high species diversity, documented acoustic behavior, and known morphological variation in the sound‐producing structures. Holocentridae (Beryciformes) meet all these criteria and therefore represent an ideal model to investigate the evolutionary history of acoustic communication mechanisms in teleosts. This family of marine tropical reef fishes (Nair and Dineshkumar 2016; Nelson 2006) is currently composed of 91 valid species (Froese and Pauly 2024; Kotlyar 2017; Matsunuma et al. 2018; Fricke 2018) that are divided into two subfamilies and nine genera (Dornburg et al. 2012): the Myripristinae (soldierfishes), composed of five genera (*Corniger*, *Pristilepis*, *Plectrypops*, *Ostichthys*, *Myripristis*) and the Holocentrinae (squirrelfishes), encompassing four genera (*Holocentrus*, *Flammeo*, *Sargocentron*, *Neoniphon*). The group likely originated near the Cretaceous–Paleogene boundary (Brownstein et al. 2025), providing a long evolutionary timespan over which sonic anatomical diversity could emerge. Many holocentrid species are reported to produce sounds in a wide variety of behaviors, both in the wild and in laboratory conditions (Banse, Bertimes, et al. 2024; Moulton 1958; Parmentier et al. 2011), such as territory defence (Winn et al. 1964), predator signaling (Mélotte et al. 2016; Winn et al. 1964; Horch and Salmon 1973; Salmon 1967), mobbing (Winn et al. 1964; Banse, Minier, et al. 2024), acoustically mediated cleaning symbiosis (Banse, Lecchini, et al. 2024) and social signaling behaviors (Banse, Hanssen, et al. 2024).

From a descriptive point of view, the sounds emitted by hand‐held *Myripristis* (Myriprististinae) specimens are generally longer, composed of more pulses, have a higher fundamental frequency and a shorter pulse period than the sounds produced by *Holocentrus*, *Neoniphon*, *Flammeo* and *Sargocentron* species (Holocentrinae). At a lower taxonomic level, a major acoustical characteristic distinguishes two groups within the genus *Myripristis* (Banse, Bertimes, et al. 2024), which align with the two distinct clades of *Myripristis* identified in the most recent phylogenetic tree of Holocentridae (Dornburg et al. 2012). One group evolved complex sound patterns characterized by unique temporal arrangements (discontinuous series of pulses, i.e., sounds composed of blocks of pulses), whereas the other group produces sounds consisting of continuous series of pulses. Such vocal ability should rely on modifications of the sound‐producing apparatus and/or the neural firing network directing the contraction of the sonic muscles (Banse, Bertimes, et al. 2024). The sound‐producing mechanism of holocentrids relies on the simultaneous contraction of paired bilateral sonic muscles (Salmon 1967), that originate on the skull and insert on the first epineurals and ribs that are in tight connection with the swim bladder (Moulton 1958; Parmentier et al. 2011; Winn and Marshall 1963). Sonic muscles are thought to derive from anterior somites, as suggested by their occipital origin and innervation by occipital nerves (Onuki and Somiya 2007; Gainer et al. 1965; Carlson and Bass 2000). Each contraction of the sonic muscle prompts a rostral displacement of the proximal end of the first ribs, thereby stretching the anterior part of the swim bladder. Although the basic sound‐producing apparatus seems to be conserved in holocentrids, some differences can be found across taxa. The main distinctions reported from the comparison of species belonging to four genera (*Holocentrus*, *Myripristis*, *Neoniphon* and *Sargocentron*) reside in: (1) the morphology of the swim bladder—composed of a main chamber and two rostral diverticula in the Myripristinae, whereas it consists in a simple tubular structure in the Holocentrinae (Nelson 1955) and (2) in the insertion of the sonic muscles, tendons and ligaments, and the number of ribs involved in the mechanism. Although not directly involved in sound production, the structural differences in sound‐producing mechanisms may be constrained by the morphology of the swim bladder, which itself directly affects hearing ability.

This study aims to investigate whether acoustic communication diversity within Holocentridae reflects their evolutionary history and how it relates to variation in the sound‐producing apparatus. To address this question, we combined phylogenetic reconstruction, morphological analysis of the sonic mechanism, and acoustic data from a broad species sample. We used Holocentridae as a model because of differences in their sound‐producing mechanism and in their sounds (Parmentier et al. 2011), their high diversity (Froese and Pauly 2024) and their huge differences in hearing abilities (Coombs and Popper 1979). We hypothesized that broad acoustic differences at higher taxonomic levels may be associated with structural differentiation of the sound‐producing system, whereas finer acoustic diversification among closely related species may occur despite substantial conservation of the sonic apparatus. By mapping morphological and acoustic traits onto a phylogenetic framework, we aim to identify whether these traits show congruent evolutionary patterns and evaluate a hierarchical scenario in which morphological differentiation and differential use of conserved anatomy both contribute to acoustic diversification.

## 2 Materials and Methods

### 2.1 Biological Material

Sampling was conducted in two phases. Initially, Holocentridae were collected by snorkeling or scuba diving in coral reef areas of Guadeloupe, French Polynesia, Guam, Seychelles, and the Philippines (Banse, Bertimes, et al. 2024). Specimens were acoustically recorded (Banse, Bertimes, et al. 2024) before being measured, both in standard and total lengths. Fish were then photographed, and fin clips sampled from the caudal fin of the specimens and stored in absolute ethanol (99%) for phylogenetic analyses before their release. Some of the specimens were, however, euthanized with an overdose of MS‐222 for morphological study and placed for 1 week in formaldehyde (5%) for fixation before being conserved in ethanol (70%).

In addition, tissue samples or DNA extracts ( *Neoniphon aurolineatus* FRLM 40009, *Myripristis kochiensis* FRLM 56669, *Ostichthys japonicus* FRLM 54433, *Ostichthys kaianus* FRLM 46469, *M. tiki* I.46467.079, *Sargocentron hormion* I.46468.023, *Pristilepis oligolepis NMNZ P.058460*, *Ostichthys japonicus* USNM 445307, *Corniger spinosus* USNM 434777/USNM 426766) and specimens ( *Beryx decadactylus* MNHN‐IC‐1956‐0039, *C. spinosus* UF 238258/UF 238259/IEOCA 898‐1, *Plectrypops retrospinis* UF 133606/UF 14086, *P. oligolepis* KPM‐NI 006833/I.32112‐001, *O. japonicus* I.25810‐001/I.25800‐001, *Ostichthys trachypoma* UF 72155) were provided by collaborators from natural history collections.

### 2.2 Phylogenetic Analysis

#### 2.2.1 Sequences

Sequences of mitochondrial DNA (mtDNA) represented by the cytochrome *c* oxidase subunit 1 (*COI*) gene and of 5 nuclear genes (*ENC1*, *Glyt*, *myh6*, *Ptr*, *sreb*) published by Dornburg et al. (2012) were downloaded from GenBank for the 40 holocentrid species these authors used in their study and for the outgroup *B. decadactylus* , a representative of the Berycidae, which is a sister clade to the Holocentridae within the Beryciformes (Near and Thacker 2024). One hundred and thirteen samples (fin clip, muscle tissue samples or DNA extracts) from our sampling in the field and museum collections were used to complement this dataset (Appendix S1). Field sampling was performed in accordance with the Nagoya protocol. The complete dataset thus comprised a total of 53 species, including the outgroup. DNA was extracted using a “NucleoSpin Tissue Kit” following the instructions of the manufacturer (Macherey–Nagel, Germany). We used polymerase chain reaction (PCR) to amplify the genes listed above.

Amplification of the mitochondrial *COI* gene fragment was conducted using an M13‐tailed primer cocktail (“Fish Cocktail”) with the primers VF2_t1, FishF2_t1, FishR2_t1, and FR1d_t1 (Ivanova et al. 2007). PCR had an initial denaturing step conducted at 94°C for 3 min; 35 cycles with a denaturation at 94°C for 45 s, annealing at 51°C for 40 s, and an extension at 72°C for 90 s, that were followed by an additional 10 min final extension at 72°C. Each 25 μL PCR mix consisted of 2.5 μL PCR buffer (10×); 2.5 μL dNTP (2 mM); 1.25 μL “Fish Cocktail” (2 μM); 0.2 μL Taq DNA Polymerase (5 units per μL); 17.75 μL mQ‐H2O and 1.0 μL of the extracted DNA sample. Nuclear genes were amplified following the methodology of Dornburg et al. (2012), using their published custom primers and a touchdown PCR strategy (Don et al. 1991). Touchdown PCR is used to minimize spurious priming: early cycles prioritize specificity under stringent conditions, while later cycles increase amplification efficiency under progressively relaxed conditions. An initial denaturing step was conducted at 94°C for 2 min, followed by 11 cycles with a denaturation at 94°C for 30 s, annealing starting at 67°C and decreasing by 1.5°C per cycle for 60 s, and an extension at 72°C for 60 s. This was followed by an additional 19 cycles with a denaturation at 94°C for 30 s, annealing at 57°C for 60 s, and an extension at 72°C for 60 s, followed by an additional final extension at 72°C for 10 min. PCR products were visually checked on 1% agarose gels and purified using an ExoSAP‐IT PCR Clean‐up Kit (Affymetrix, Cleveland, USA). All procedures were carried out at the Royal Belgian Institute of Natural Sciences (RBINS). Purified PCR products were sent to Macrogen Inc. for Sanger sequencing.

#### 2.2.2 Alignment

The DNA sequences were assembled and visually checked in CodonCode Aligner v. 8.0.2 (CodonCode Corporation). Alignments were done using Muscle Alignment (Edgar 2004) in MEGA 11.0.8 (Tamura et al. 2021) and checked by eye. Sequences were trimmed to match the size of the downloaded fragments for each gene. The final alignments consisted of a total of 4015 base pairs (bp): *COI* (651 bp), *ENC1* (695 bp), *Glyt* (721 bp), *myh6* (534 bp), *Ptr* (561 bp), and *sreb* (853 bp).

#### 2.2.3 Likelihood and Bayesian Phylogenetic Inference

Seven datasets were first analyzed: one for each gene and a concatenated matrix of the five sampled nuclear genes. Because the trees generated from individual genes, the concatenated nuclear dataset, and the mitochondrial *COI* gene were similar, we inferred an additional tree using the concatenated matrix of these sequences.

All genes were partitioned with each position in a codon as an individual partition. Phylogenetic trees were constructed using the maximum likelihood (ML) method and Bayesian inference (BI) (Yang 1994; Huelsenbeck and Ronquist 2001). Bayesian inference of phylogeny is based on a quantity called the posterior probability distribution of trees, which is the probability of a tree conditioned on the observations (Lin and Hastings 2011), whereas the principle of the maximum likelihood lies in the construction of a tree that makes the observed data “most likely” (Myung 2003). Nucleotide substitution models (Edge‐linked; with FreeRate heterogeneity) were selected (Appendix S2) and the ML phylogenies inferred using IQ‐TREE (Trifinopoulos et al. 2016; Nguyen et al. 2015; Chernomor et al. 2016) under the Edge‐linked partition model for 1000 ultrafast bootstraps (Hoang et al. 2018). The BI phylogenies were inferred using MrBayes v3.2.7a (Ronquist et al. 2012) on the Cipres Portal (Miller et al. 2010) with two parallel independent runs starting from different random trees and 10,000,000–60,000,000 generations. The number of generations was set to 10,000,000 and increased by 10,000,000 if convergence (average standard deviation of split frequencies < 0.005) was not reached. For the tree obtained from both the mitochondrial and nuclear gene matrix, we performed a “Simpler” analysis where partitions for which models were identical were gathered. For this analysis, the number of generations was set to 20,000,000. Convergence was further checked in Tracer v1.7.2. Majority‐rule consensus phylogenetic trees were generated after discarding the initial 25% of the sampled data which were considered as burn‐in. TreeGraph v2.15.0‐887 beta (Stöver and Müller 2010) was used to visualize and edit the phylogenetic trees.

The feasibility of combining phylogenetic information based on the five nuclear and the mitochondrial markers has been evaluated by looking for strongly supported but conflicting clades (Wiens 1998). Clades were considered strongly supported if the bootstrap support or the posterior probability was ≥ 95% in the ML and BI analyses.

#### 2.2.4 Species Tree Inference

A species tree was additionally estimated using multispecies coalescent Bayesian inference with StarBEAST2 (v.2.7.7) (Heled and Drummond 2010). The six gene alignments (*COI*, *ENC1*, *Glyt*, *myh6*, *Ptr* and *sreb*) were treated as unlinked partitions. The analysis was conducted using the random local clock method (Drummond and Suchard 2010), and the Yule prior for speciation rate. Markov chain Monte Carlo (MCMC) chains were run for 60 million generations. Convergence was assessed in Tracer v1.7.2. (Rambaut et al. 2018), and the first 25% of samples were discarded as burn‐in. A maximum clade credibility tree was then generated from the posterior distribution.

### 2.3 Morphology of the Sound‐Producing Mechanism

The study of the sound‐producing mechanism focused on 17 holocentrid species (Appendix S3). Species were selected to encompass most branches of the phylogenetic tree of Holocentridae. Additionally, we investigated the morphology of the neurocranium, skeleton, and swim bladder of the outgroup *B. decadactylus*. Key morphological terminology used in this study is defined in Appendix S5, and these terms are indicated in bold at their first occurrence in the text.

Investigations of the sound‐producing mechanisms were first based on 3D reconstructions from μCT scans of the specimens. Fifteen specimens were stacked scanned (multiple rotations per full scan) using an EasyTom 150 (RX Solutions, Chavanod, France, https://www.rx‐solutions.com/) with an aluminum filter at 110 kV and 1440 projections per rotation at the Royal Belgian Institute of Natural Sciences (RBINS) in Brussels, Belgium. For these specimens, the isotropic voxel resolution varied between 19.7 and 51.6 μm (μm). *Beryx decadactylus* was scanned with a Phoenix v|tome|x L240‐180 (Waygate Technologies) with an aluminum filter at 140 kV and 2250 projections per rotation at the Muséum National d'Histoire Naturelle (MNHN) in Paris (France) at a voxel resolution of 85.1 μm. *Corniger spinosus* was scanned using a TESCAN UniTOM XL at the University of Antwerpen, Belgium, with an aluminum filter at 60 kV and 2217 projections per rotation, at a voxel resolution of 73.8 μm. Micro‐CT scans of *Myripristis jacobus* (specimen ID: 000S21071) were downloaded from www.MorphoSource.org. Three morphological structures of interest (neurocranium, skeleton and swim bladder) could be reconstructed using the software AMIRA v.6.2.0. The description of additional structures (muscles, tendons and ligaments) involved in sound production required the dissection and examination of one to three specimens per species (for 11 selected species; Appendix S3) using a Wild M10 binocular microscope (Leica Microsystems GmbH, Germany) equipped with a camera lucida. These were then modeled in Blender v.2.79, using polygonal meshes that were manually shaped and adjusted to match the anatomical positions observed during dissections and aligned with the μCT‐derived skeletal and swim bladder structures reconstructed in AMIRA, exported as .ply files and imported into Blender. These reconstructions were used for qualitative anatomical comparisons of the sound‐producing apparatus across species. The images of the whole specimens and the 3D models of the sound‐producing apparatus are available on the online repository MorphoSource (https://doi.org/10.17602/M2/L896752).

### 2.4 Sound Production

The acoustic dataset used here was previously collected and thoroughly analyzed in Banse, Bertimes, et al. (2024). In short, 388 holocentrid specimens from 33 species belonging to five genera within the Myripristinae (11 *Myripristis* species) and the Holocentrinae ( *Flammeo marianus* , 11 *Sargocentron*, 8 *Neoniphon* and 2 *Holocentrus* species) were used. For each individual, 20 sounds were recorded while hand‐held, and 10 temporal and frequential acoustic features were measured. Four variables (sound duration, number of pulses in the sounds, duration of the last pulse and dominant frequency) were size‐corrected, as individual size influences these parameters (Banse, Bertimes, et al. 2024). Univariate and multivariate statistical analyses, including principal component analyses, were previously conducted on this dataset by Banse, Bertimes, et al. (2024). The present study builds on these validated results and does not repeat these analyses; full methodological details are available in the cited paper.

Additional recordings of *O. trachypoma* were performed in August 2025 at the Okinawa Churaumi Aquarium, using the same methodology, except that this species was recorded in a tank. These recordings confirm the sound‐producing ability of species in this genus.

### 2.5 Ancestral Trait Mapping

Morphological and acoustic traits were mapped on the species tree using package Phytools v.2.5.2 (Revell 2024) after pruning the tree according to species trait availability. Ancestral states for continuous acoustic traits were estimated using maximum likelihood under the Brownian motion model (Felsenstein 2004) with the Phytools function fastAnc following Revell and Harmon (2022). For all acoustic traits considered here, the Brownian motion model fitted our data better than the “early burst” (EB) (Blomberg et al. 2003), and the Ornstein–Uhlenbeck (OU) (Hansen 1997) models on the basis of the Akaike Information Criterion (AIC) (Akaike 1974). For morphological discrete traits, posterior probabilities of ancestral states were estimated using stochastic character mapping (Huelsenbeck et al. 2003) under the equal‐rates (ER) model. This model was selected for each trait following comparisons with the symmetric transition (SYM) and the all‐rates‐different (ARD) models based on the AIC (Revell and Harmon 2022).

## 3 Results

### 3.1 Phylogeny

#### 3.1.1 Likelihood and Bayesian Phylogenetic Inference

**FIGURE 1:**
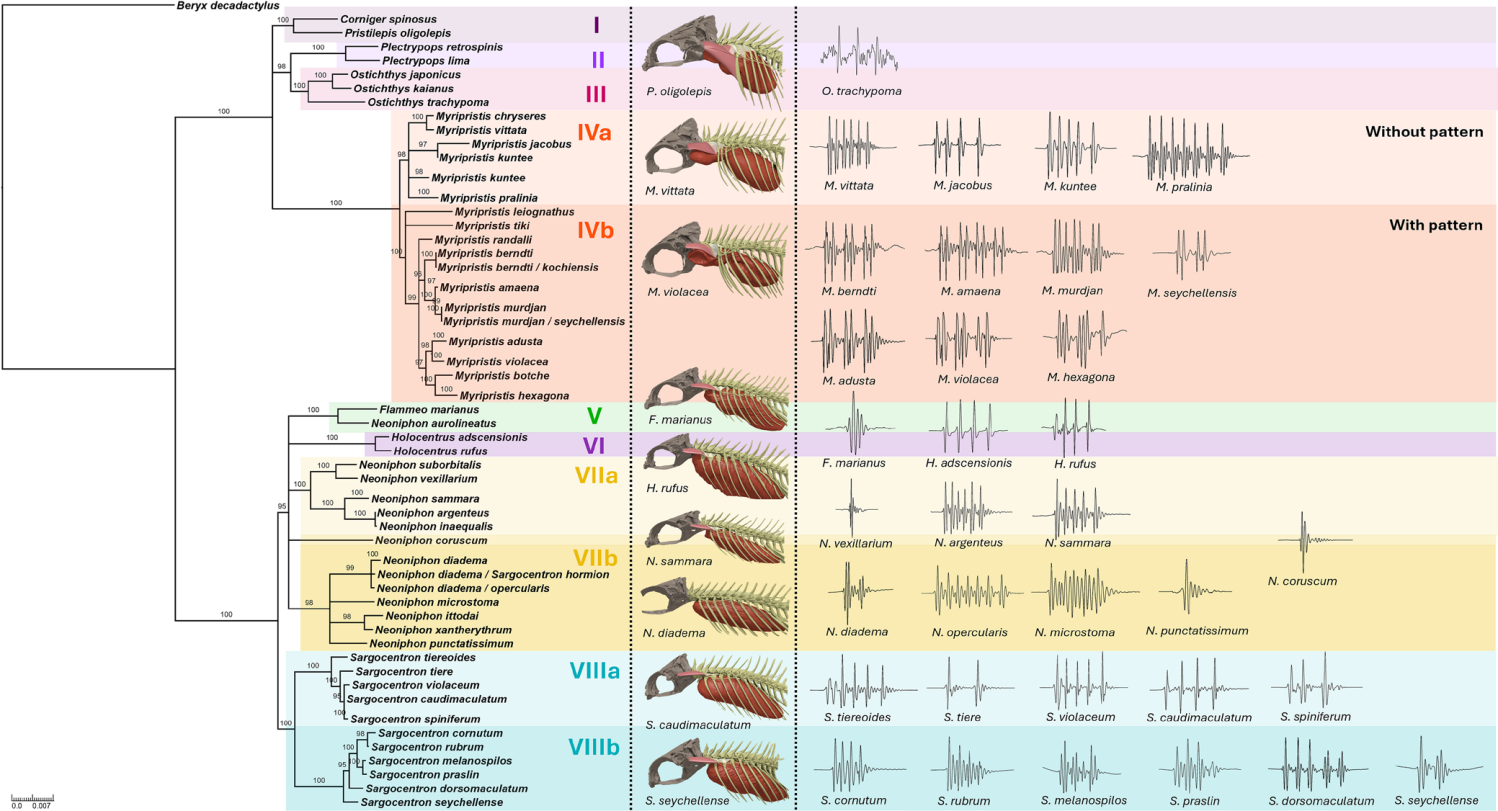
Phylogenetic relationships and acoustic diversity within Holocentridae. Maximum‐likelihood phylogeny of 52 holocentrid species and the outgroup *Beryx decadactylus* (Berycidae), based on a concatenated matrix of six nuclear and mitochondrial genes. Numbers at nodes indicate bootstrap support (%); only values ≥ 95%, considered well supported, are shown. Monophyletic species‐level clades and branches with bootstrap support < 95% were collapsed. Three‐dimensional reconstructions of the sonic apparatus are mapped for selected species, together with representative calls from all recorded taxa, illustrating morphological and acoustic affinities across lineages. Sounds have been ordered according to the phylogenetic tree.

Best‐fitting substitution models varied across partitions and genes. For both the concatenated nuclear gene (cnDNA) matrix and nuclear and mitochondrial gene (mtDNA) matrix, all gene partitions were combined in a 15 and 18‐partitions set‐up, respectively (Appendix S2). While no discordance was detected between ML and BI inferences, three small conflicts were detected between the phylogenies resulting from the *COI* and the nuclear datasets (hereafter detailed). The ML tree constructed with the mitochondrial and nuclear markers is presented in Figure 1 (uncollapsed trees are depicted in Appendices S4.1–S4.2). All other trees can be found in Appendices S4.3–S4.16. Posterior probabilities of BI analyses were mostly consistent with the bootstrap values of the ML analyses. Therefore, the groupings presented below are supported by both approaches, unless stated otherwise.

#### 3.1.2 Assessment of the Monophyly of the Subfamilies and Genera

The analysis of the cnDNA and mtDNA dataset resulted in a well resolved phylogeny supporting the monophyly of both the Myripristinae and the Holocentrinae (Figure 1). Similarly, within the Myripristinae, the monophyly of all genera is well resolved. Within the Holocentrinae, the situation is more complex. While the monophyly of *Holocentrus* is well resolved, *Neoniphon* and *Sargocentron* are resolved as polyphyletic (Figure 1). Indeed, *Holocentrus* spp., *Flammeo*, and *S. hormion* are nested within *Neoniphon*. Additionally, *Neoniphon aurolineatus* is resolved as a sister species of *F. marianus* .

#### 3.1.3 Analysis of the Phylogenetic Relationships

The analysis of the phylogeny supports the existence of three primary clades within the Myripristinae (Figure 1): (I) one comprising *Corniger* and *Pristilepis*; (II) the second one clustering *Plectrypops* with *Ostichthys*; and (III) the third one encompassing all *Myripristis*. *Myripristis* can be subdivided into two smaller clades: (IVa) *Myripristis* of group 1 ( *M. kuntee* , *M. pralinia* , *M. jacobus* , *M. vittata* and *M. chryseres* ) and (IVb) *Myripristis* of group 2 ( *M. leiognathus* , *M. tiki* , *M. randalli* , *M. berndti* , *M. kochiensis* , *M. murdjan* , *M. amaena* , *M. seychellensis* , *M. hexagona* , *M. botche* , *M. violacea* and *M. adusta* ). Note that within clade IVb, *M. leiognathus* and *M. tiki* (all markers available and five out of six markers available, respectively; Appendix S1) are sister species of all other species belonging to that clade. Within the Holocentrinae, six subclades are resolved: (V) *F. marianus* + *N. aurolineatus* , (VI) *Holocentrus* spp., (VIIa) *Neoniphon* of group 1 (*N. suborbitalis*, *N. vexillarium*, *N. sammara* , *N. argenteus*, and *N. inaequalis* ), (VIIb) *Neoniphon* of group 2 (*N. ittodai*, *N. xantherythrum*, *N. punctatissimum*, *N. microstoma* , *N. diadema*, *N. opercularis* ) + *S. hormion* , (VIIIa) *Sargocentron* of group 1 ( *S. tiere* , *S. tiereoides*, *S. violaceum* , *S. caudimaculatum*, and *S*. *spiniferum*) and (VIIIb) *Sargocentron* of group 2 ( *S. cornutum* , *S. dorsomaculatum* , *S. melanospilos* , *S. praslin* , *S. rubrum*, and *S. seychellense* ). The position of *N. coruscum* is not resolved within the *Neoniphon*.

Note that the position of *N. punctatissimum*, *S. rubrum* , *S. tiere* , and *S. tiereoides* vary between the topologies obtained from the mtDNA and cnDNA analyses (Appendices S4.3–S4.6; species indicated with an asterisk in Figure 1).

The analyses performed using the mtDNA *COI* gene resulted in a tree topology (Appendices S4.5–S4.6) that is similar to that obtained using the cnDNA (Appendices S4.3–S4.4), with a few exceptions. (1) *Neoniphon punctatissimum* clusters with clade VIIb of *Neoniphon* in the cnDNA phylogeny while it clusters with clade VIIa of *Neoniphon* in the mtDNA phylogeny. (2) *Sargocentron rubrum* is a sister species of *S. melanospilos* and *S. praslin* in the cnDNA phylogeny while it clusters with *S. cornutum* in the mtDNA phylogeny. (3) Within clade VIIIa, *Sargocentron tiere* is a sister species of the remaining *Sargocentron* species in the cnDNA phylogeny while *S. tiereoides* is the sister species of the remaining *Sargocentron* species of this clade in the mtDNA phylogeny.

#### 3.1.4 Species Tree

A species tree inferred under multispecies coalescent framework is provided in Appendix S4.17. This analysis yielded a topology largely consistent with those obtained from the concatenated nuclear and mitochondrial matrix, with no major differences affecting the interpretation of phylogenetic relationships.

### 3.2 Morphology of the Sound‐Producing Mechanism

**FIGURE 2:**
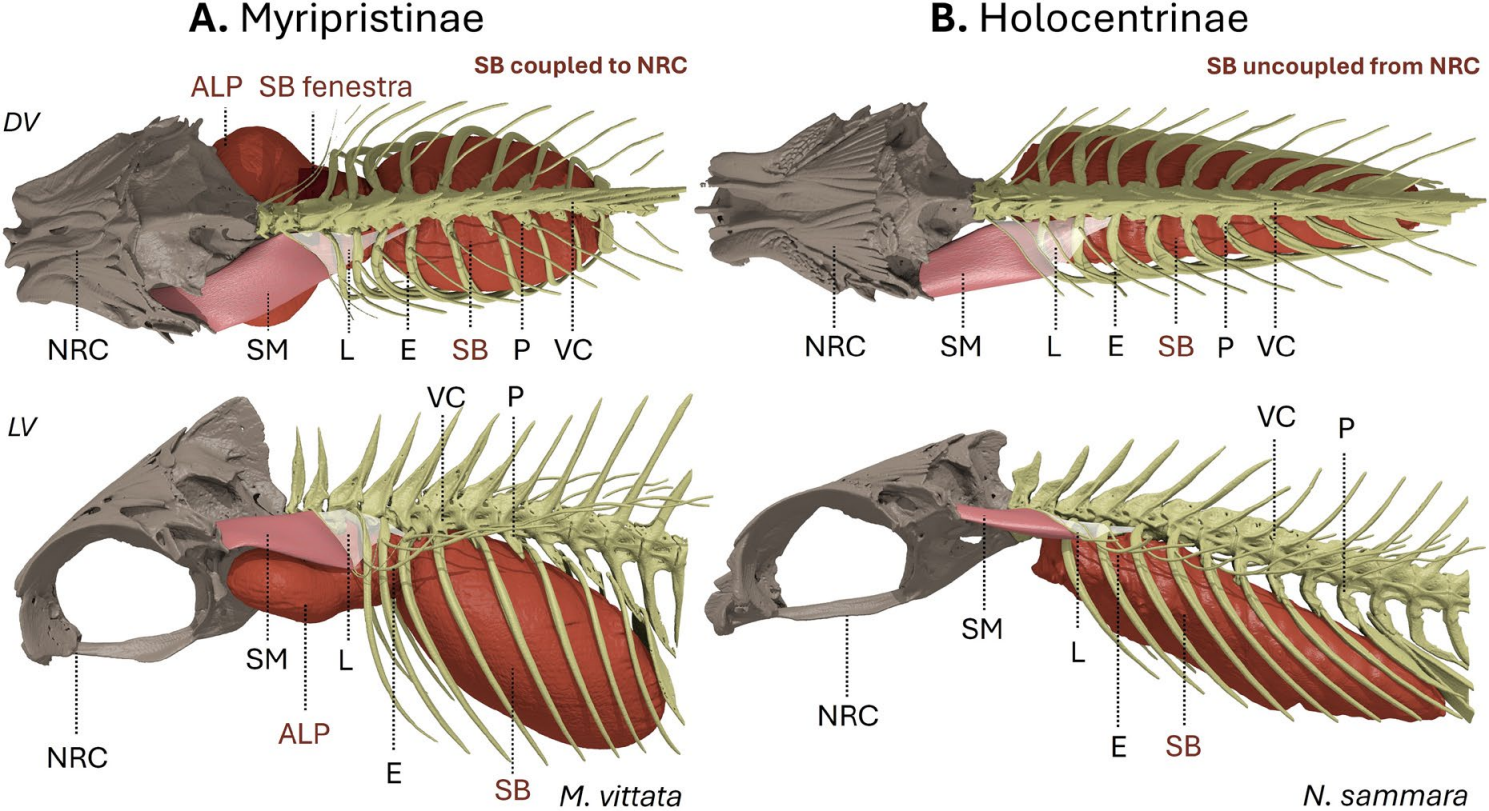
Dorsal (DV) and left lateral (LV) views of the sonic apparatus in (A) Myripristinae ( *Myripristis vittata* ) and (B) Holocentrinae ( *Neoniphon sammara* ). In dorsal view, only the left sonic muscles (SM) and ligaments (L) are depicted. The swim bladder (SB) fenestra is therefore visible on the right side only since it is hidden by the sonic muscles and ligaments on the left side. ALP, antero‐lateral projections; E, epineurals; NRC, neurocranium; *P*, parapophyses; VC, vertebral column.

**FIGURE 3:**
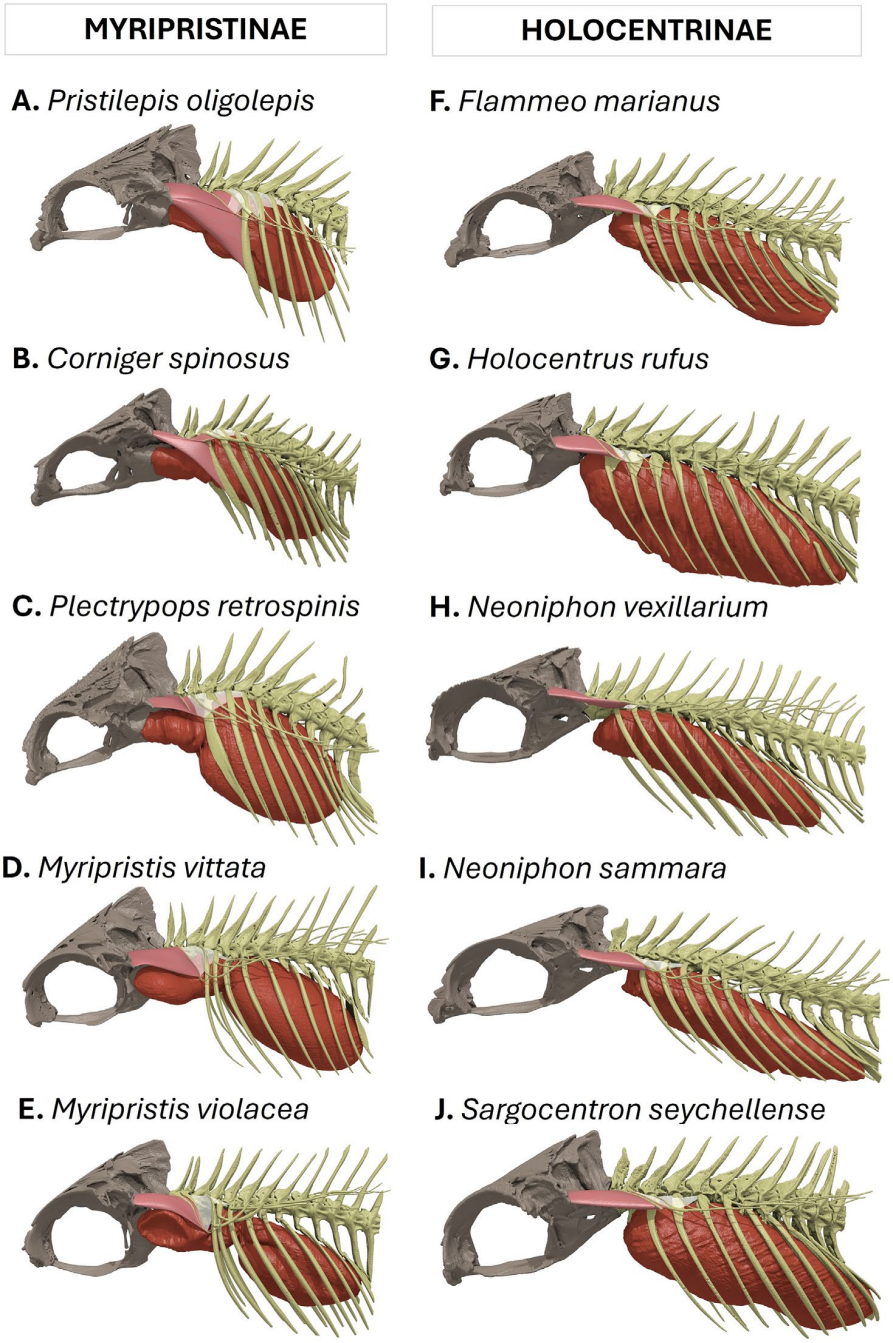
Left lateral view (3D reconstruction) of the sound‐producing mechanism in five Myripristinae species (A–E) and five Holocentrinae species (F–J).

In all holocentrids, the sound‐producing mechanism involves the skull, the axial skeleton, the swim bladder, and bilateral sonic muscles (Figures 2 and 3). For all species, the mechanism is based on the same principle: high‐speed sonic muscles originate on the posterior part of the neurocranium and insert on the first epineurals and/or ribs that are intimately connected to the dorsal part of the swim bladder walls. Although the main components are consistent across taxa, organizational differences can be found in the axial skeleton, swim bladder shape, and its connection to the neurocranium and vertebrae, and in the organization of muscles and ligaments.

#### 3.2.1 Skull

**FIGURE 4:**
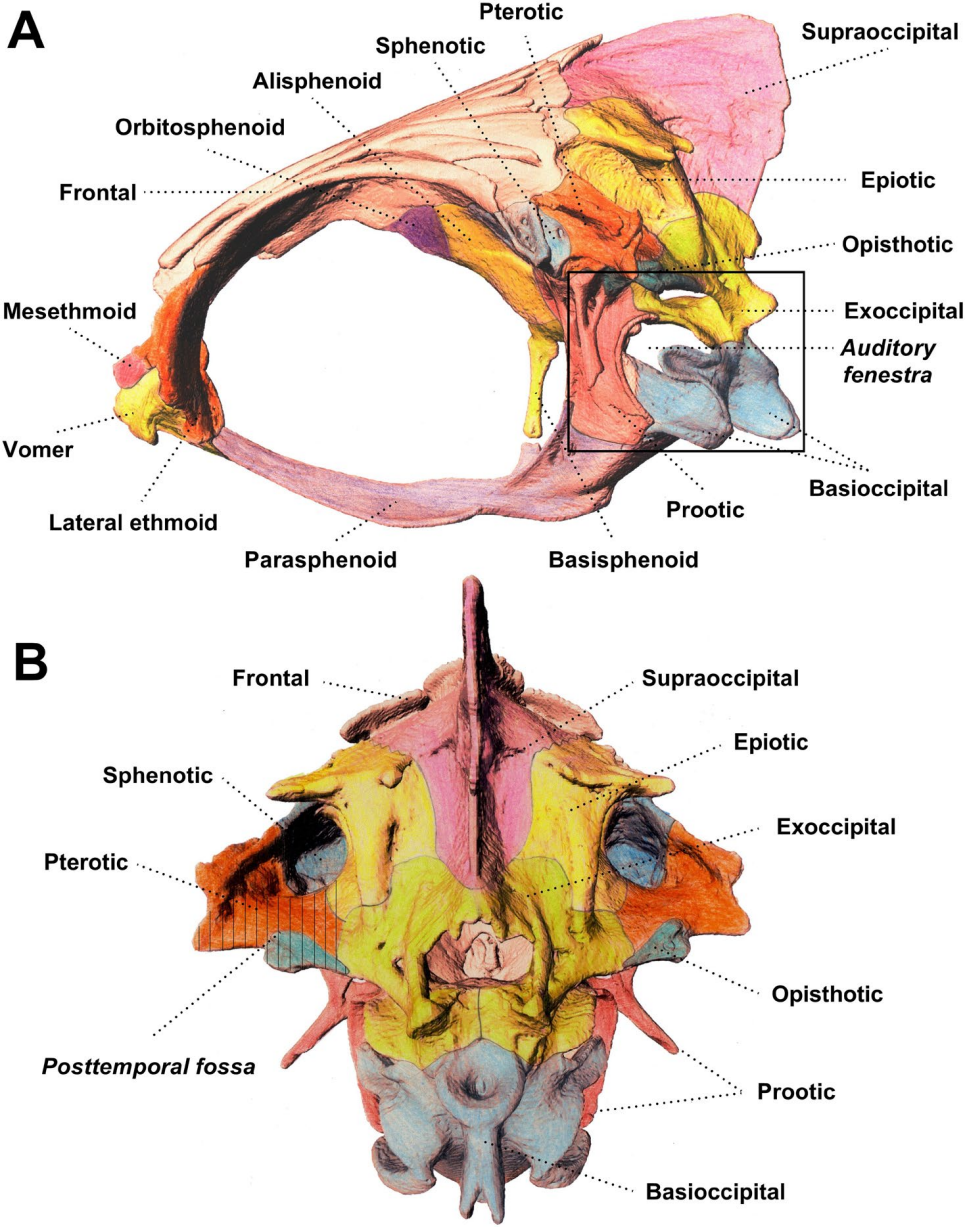
Reconstruction in 3D of the neurocranium of *Myripristis violacea* (Myripristinae) in lateral (A) and caudal (B) views, with different colors indicating the different cranial bones. The surface constituted by part of the pterotic, epiotic, and opisthotic bones forms the *posttemporal fossa*. The rectangle indicates the position of the *auditory bulla* that possesses a membranous *auditory fenestra* enclosed by the prootic, exoccipital, and basioccipital.

**FIGURE 5:**
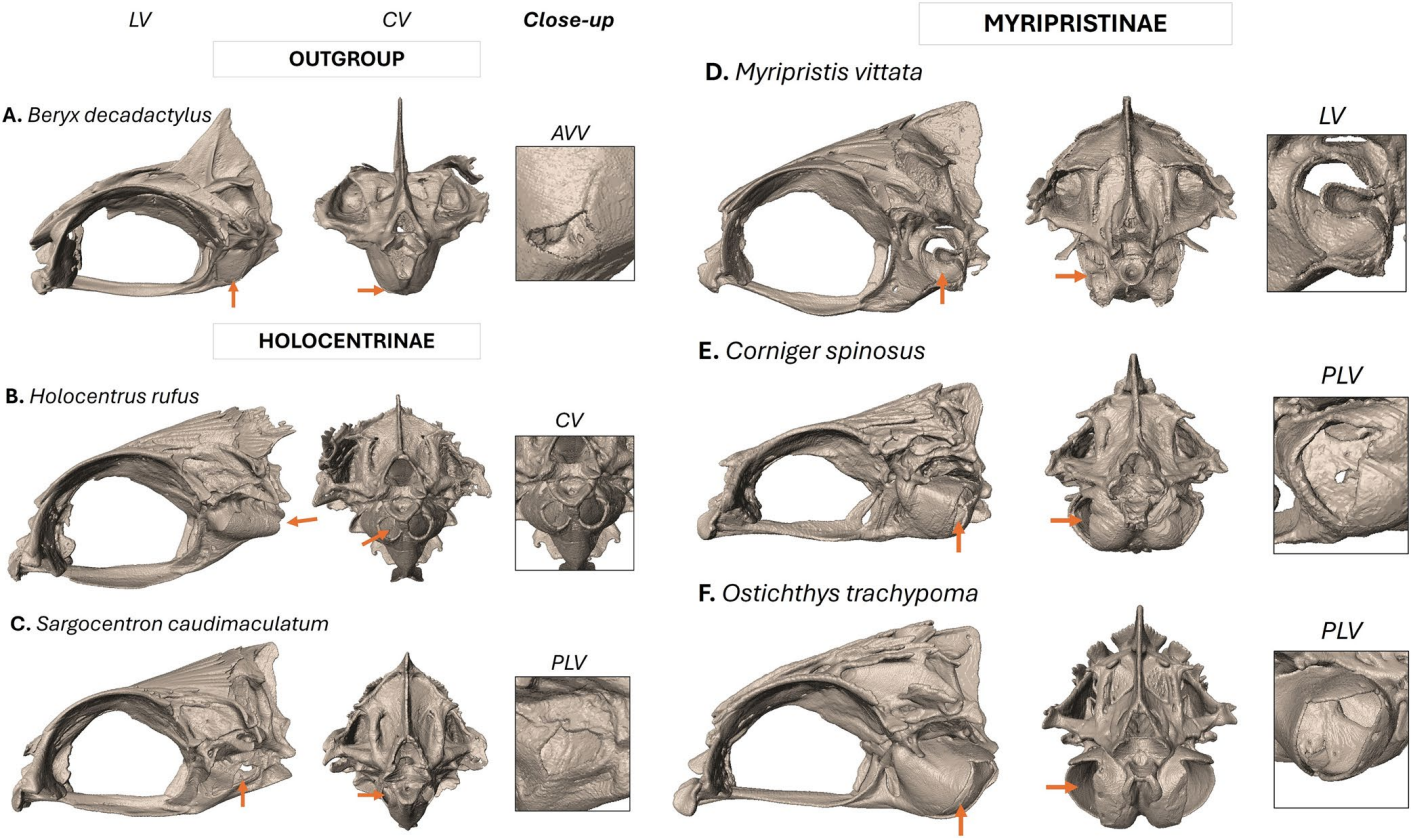
Left lateral (LV) and caudal (CV) views (3D reconstructions) of the neurocranium in *Beryx decadactylus* and several holocentrid species showing the variation in position and shape of the auditory fenestra (indicated by the arrows) across taxa. (A) antero‐ventral and triangular, (B) caudal and circular, (C) postero‐lateral and curvilinear triangular, (D) lateral and ear‐shaped, (E) postero‐lateral and semi‐circular, and (F) postero‐lateral and elliptical. The close‐ups consist of the enlargements of the membranous areas (not shown) of the auditory bulla (B–F). The position and shape of the auditory bulla reflect the organization of the belonging genus. The organization displayed in C and E additionally reflects that of *Flammeo* and *Neoniphon* species, and *Plectrypops* and *Pristilepis* species, respectively. AVV, antero‐ventral view; PLV, postero‐lateral view.

In the otic part of the skull, the auditory fenestra corresponds to an area devoid of bones, but laterally covered by connective tissue, and facing the pars inferior of the inner ear (Figures 4 and 5). In all species, the auditory fenestra is enclosed by the prootic, exoccipital, and basioccipital bones (Figures 4 and 5), but it varied in position and shape across genera (Appendix S6).

The otic region of *B. decadactylus* possesses a small auditory fenestra located antero‐ventrally on the otic region (Figure 5A). Within the Holocentrinae, the auditory fenestra of *Flammeo*, *Sargocentron*, and *Neoniphon* species is similar in shape to that of *B. decadactylus* but appears to be proportionally wider (Figure 5C). *Holocentrus* distinguishes from all other holocentrid genera by an elongated and curved tubular otic region enclosing a circular auditory fenestra caudally (Figure 5B). Within Myripristinae, *Myripristis* species differ from all other myripristine genera by the ear‐shaped morphology of the auditory fenestra, resulting from a lateral projection of the basicoccipital (Figure 5D). In *Corniger*, *Plectrypops*, and *Pristilepis* species, the auditory fenestra is semi‐circular in shape (Figure 5E), whereas in *Ostichthys* it is more elliptic and proportionally wider (Figure 5F).

#### 3.2.2 Vertebral Column

**FIGURE 6:**
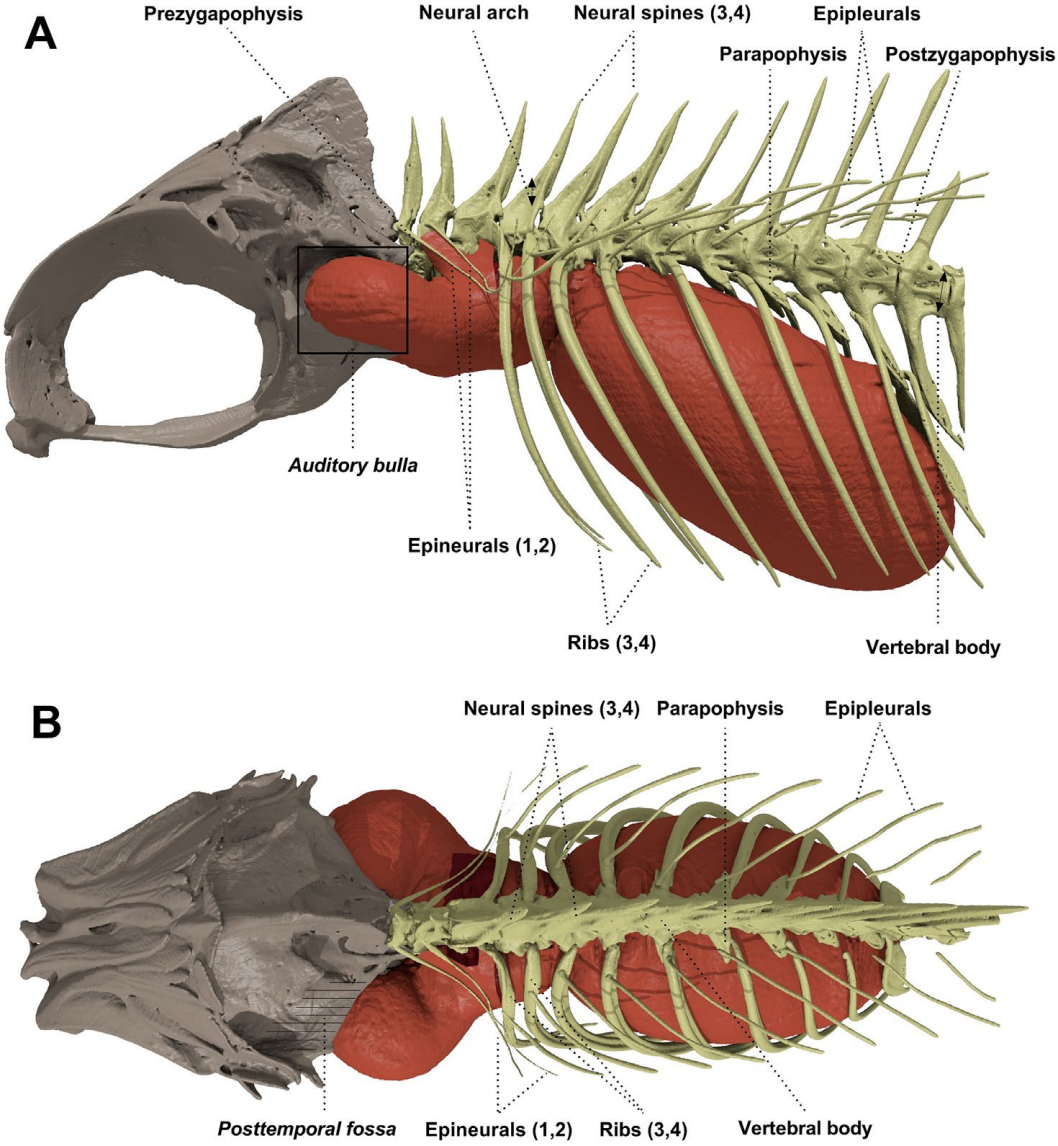
3D Reconstruction of the neurocranium, axial skeleton and swim bladder of *Myripristis vittata* (Myripristinae) in (A) lateral and (B) dorsal views. The rectangle indicates the position of the *auditory bulla* that possesses a membranous *auditory fenestra* (hidden by the rostral diverticula of the swim bladder).

**FIGURE 7:**
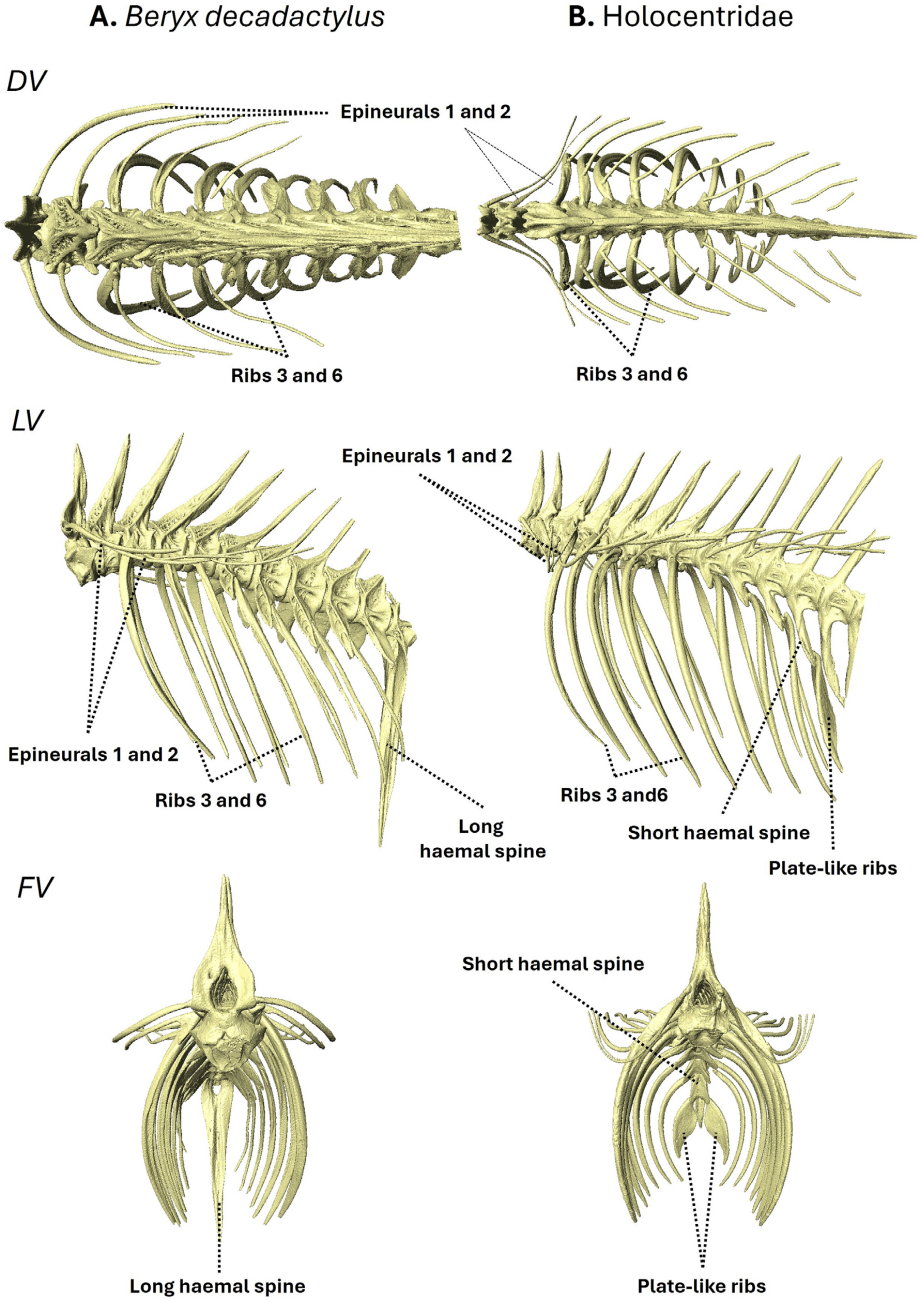
Dorsal (DV), lateral (LV), and frontal (FV) views (3D reconstructions) of the morphology of the anterior part of the vertebral column in (A) *Beryx decadactylus* and (B) *Myripristis vittata* , as a representative of holocentrid species.

Depending on the species, 11–14 vertebrae surmount the abdominal cavity. In *B. decadactylus* and all Holocentridae, the first two vertebrae possess epineurals that articulate at the level of the neural arch (Figures 6 and 7). From vertebra III to XII, the epipleurals articulate at the level of the ribs or vertebral bodies (Figure 6). Depending on the vertebra, the ribs articulate either directly on the vertebral body or at the level of the parapophyses, whose presence and size vary with species (Figures 6 and 7). In *Beryx decadactylus* , the haemal arch closes at vertebra XI and is continued by a long vertical haemal spine that delineates the caudal part of the body cavity (Figure 7; Appendix S7). In holocentrid species, the haemal arch closes earlier (vertebra VIII–IX), with a shorter haemal spine having articulated plate‐like ribs that delineate the posterior part of the body cavity (Appendix S7). Ribs are uniform in *B. decadactylus* , with ribs 3–5 attaching to vertebrae lacking parapophyses and rib 6 articulating with the first parapophysis on vertebra VI. Within Holocentridae, several skeletal characteristics distinguish taxa at the genus and species levels (Appendix S7).

**FIGURE 8:**
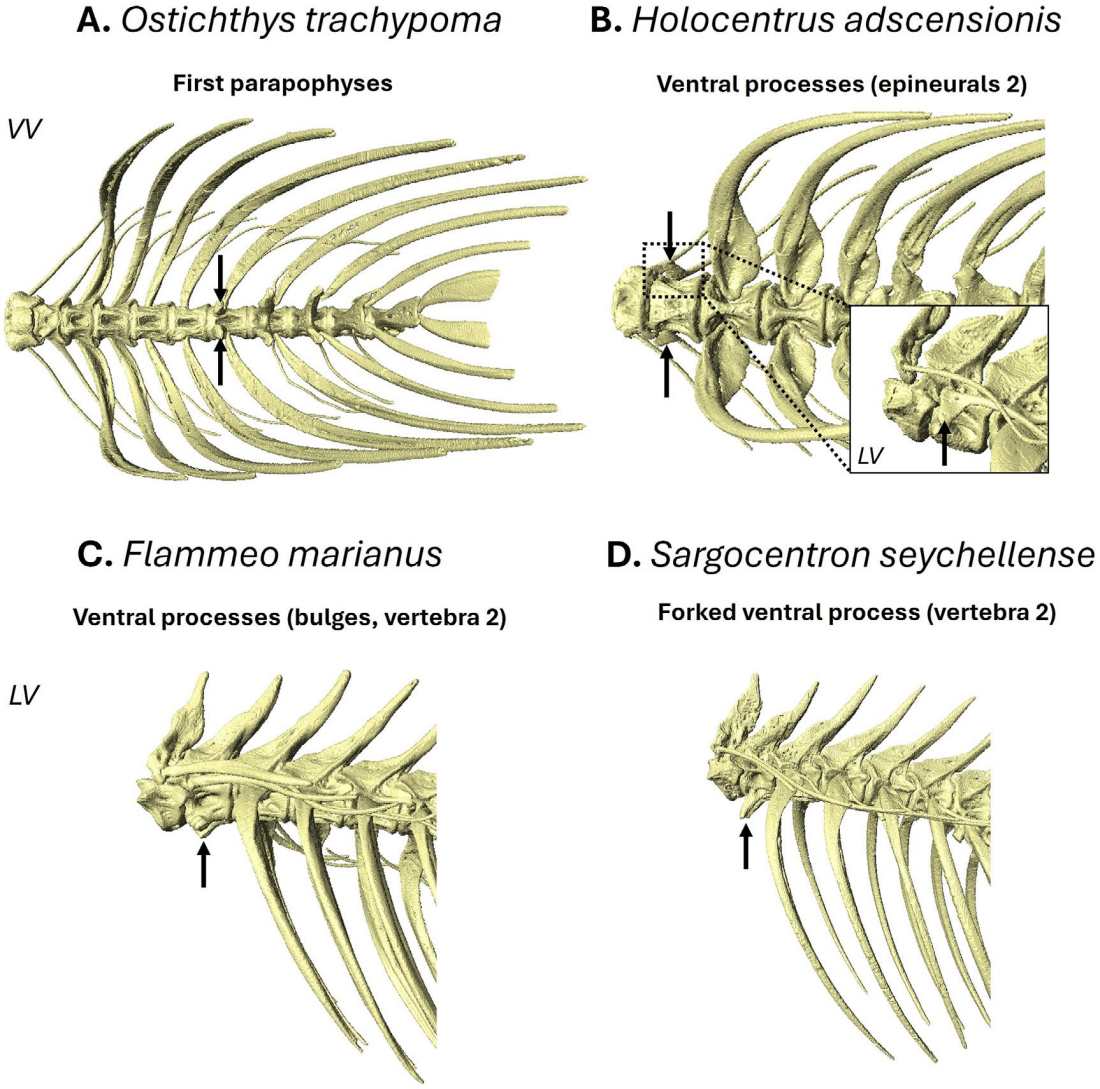
3D Reconstructions of the axial skeleton in (A) *Ostichthys trachypoma* , (B) *Holocentrus adscensionis* , (C) *Flammeo marianus* and (D) *Sargocentron seychellense* . (A) The first parapophyses are found on vertebra 7 in Ostichthys, Corniger, Pristilepis and Plectrypops, whereas in Myripristis, it depends on the species (5 or 6). In the Holocentrinae (*Flammeo*, *Holocentrus*, *Neoniphon* and *Sargocentron*), the first parapophyses are found on vertebra 6. (B) *Holocentrus* species possess a ventral process at the base of the 2nd epineurals. (C) *Flammeo marianus* is characterized by the presence of two ventral processes that have the aspects of small bugles below vertebra 2. (D) Sargocentron species are characterized by the presence of a forked ventral process projecting forward below vertebra 2. LV, lateral view; VV, ventral view.

Within the Myripristinae, the first parapophyses are found on vertebra VII in *Corniger*, *Pristilepis*, *Plectrypops*, and *Ostichthys* (Figure 8A, Appendix S7), but on vertebra V or VI in *Myripristis*. Within the Holocentrinae, they appear on vertebra VI. *Holocentrus* species are distinguished by a ventral process at the base of the 2nd epineural (Figure 8B). *Flammeo marianus* shows two small bulges below vertebra II (Figure 8C), whereas *Sargocentron* species possess a forked ventral process below vertebra II (Figure 8D).

**FIGURE 9:**
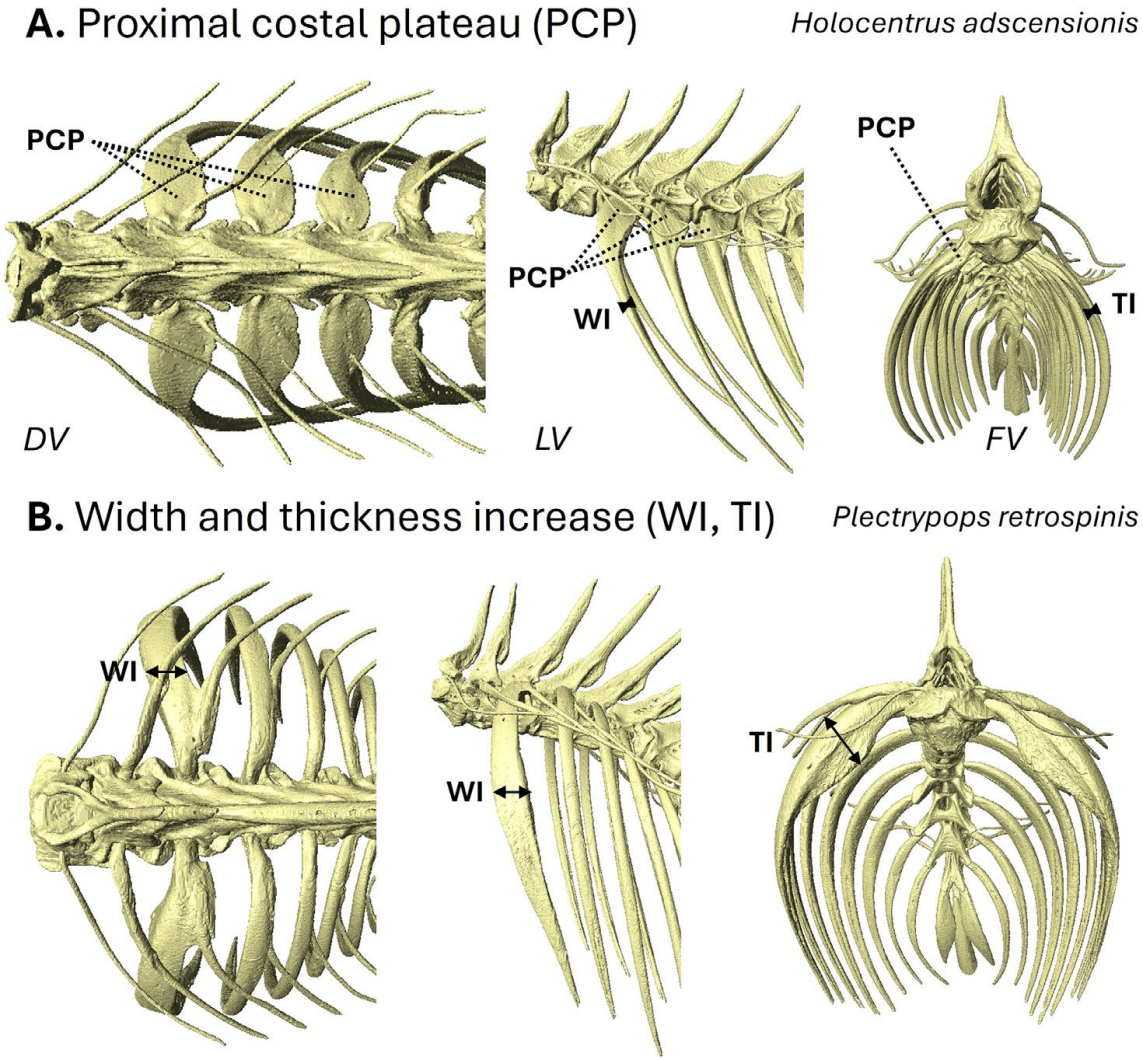
Dorsal (DV), lateral (LV) and frontal (FV) views (3D reconstructions) of the two main types of morphological variations of the ribs observed across holocentrid taxa in this study. (A) Presence of a proximal costal plateau (PCP on ribs 3, 4 and 5 in *Flammeo*, *Holocentrus* and *Sargocentron* species). (B) Increase of the width (WI) and thickness (TI) of rib 3 in *Corniger*, *Plectrypops*, *Ostichthys*, and *Pristilepis*. In *Neoniphon*, ribs do not show any particularities in shape.

Our comparison focuses on the skeletal structures associated with the sound‐producing mechanism, which show clear interspecific differences (Appendix S7). In Holocentrinae, ribs 3–5 articulate in a horizontal plane with vertebrae lacking parapophyses, while rib 6 attaches to the first parapophysis on vertebra VI. These first ribs possess laterally expanded bases forming a proximal costal plateau (PCP) particularly developed in *Holocentrus* and *Sargocentron*, but proportionally narrower in *Flammeo* (Figures 8B–D and 9A). From vertebra VI, the ribs no longer have this appearance and are uniform in width and height. In *Neoniphon* species only, ribs 3–6 do not show that distinctive morphology.

In all Myripristinae, the 3rd rib articulates in a vertical plane on the neural arch, resulting in a dorsal rib portion that is thicker than wide. In *Corniger*, *Pristilepis*, *Ostichthys*, and *Plectrypops*, the 3rd rib is relatively wider and thicker than more posterior ribs, a feature particularly pronounced in *Plectrypops* (Figure 9B). In these genera, ribs 4 and 5 also articulate on the neural arch but are comparatively narrower, whereas rib 6 inserts directly on the 6th vertebral body. In *Myripristis*, *M. violacea* has ribs 3–5 articulating on the neural arch and rib 6 on the first parapophyse, whereas ribs 3 and 4 articulate on the neural arch, and ribs 5 and 6 on the parapophyses in *M. vittata* , *M. berndti* , and *M. jacobus* .

#### 3.2.3 Swim Bladder

**FIGURE 10:**
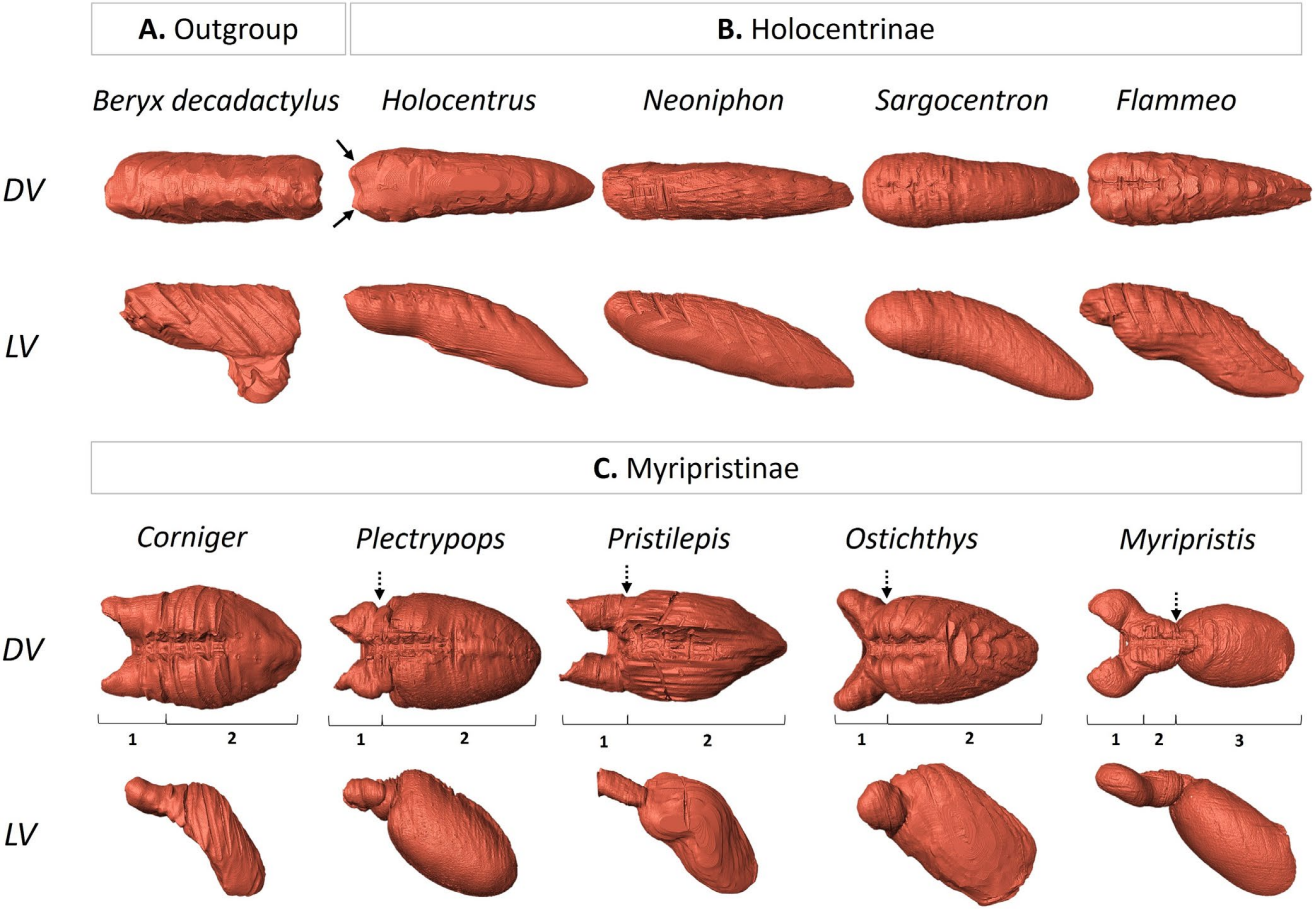
Morphology of the swim bladder in *Beryx decadactylus* (A) and the holocentrid genera within the Holocentrinae (B) and the Myripristinae (C). DV, dorsal view; LV, lateral view. The dotted arrows indicate the constriction zone on the swim bladder. The numbers correspond to the different parts of the swim bladders. The plain arrows indicate the antero‐lateral bulges in *Holocentrus*.

**FIGURE 11:**
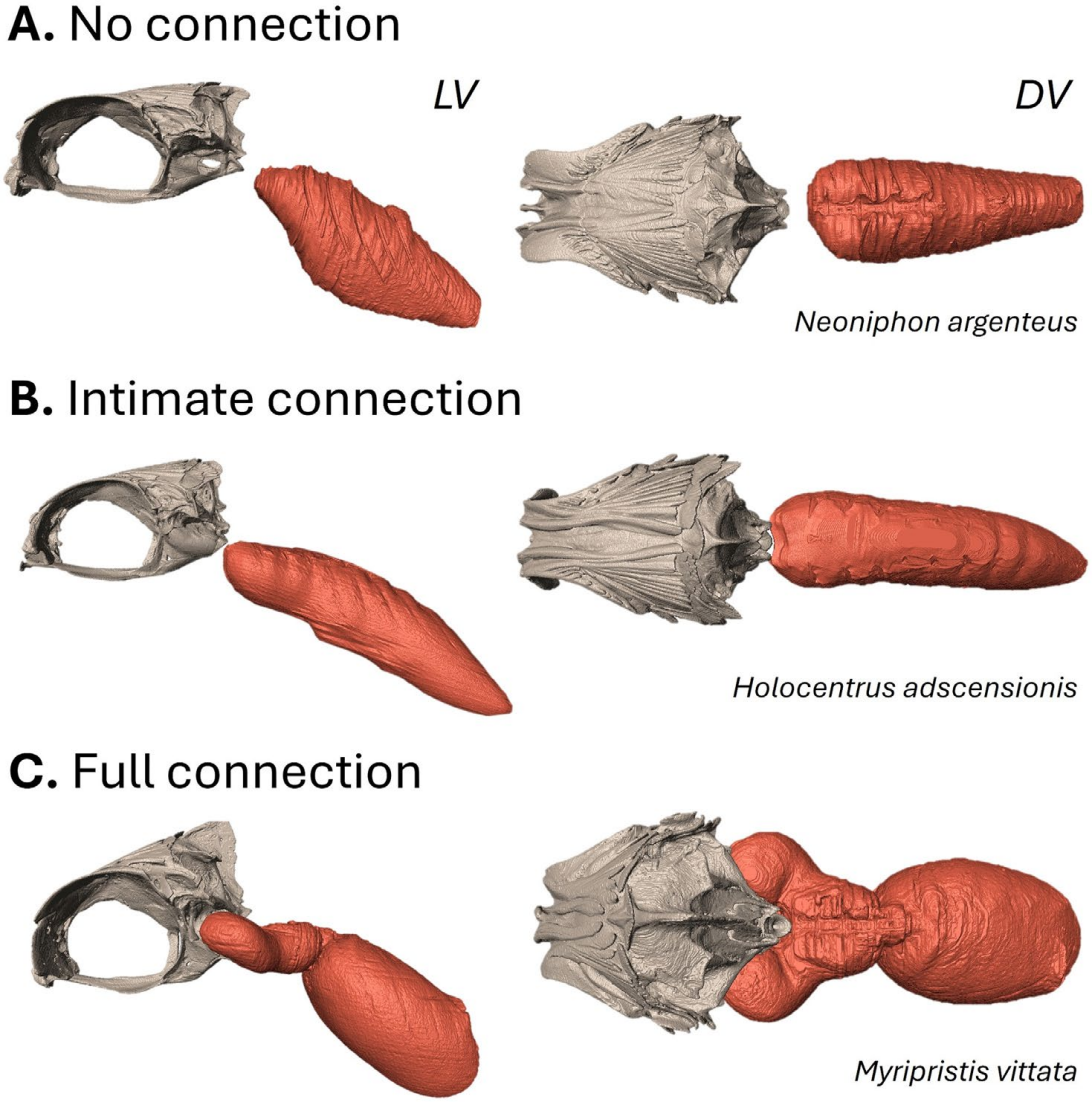
Summary of the three distinct types of connections existing between the neurocranium and the swim bladder in holocentrid species. (A) There is a lack of connection between the neurocranium and the swim bladder in *Neoniphon*, *Flammeo*, and *Sargocentron*. (B) The connection of the swim bladder with the membranous area of the auditory bullae is intimate but limited to the antero‐lateral bulges on the caudal end of the skull in *Holocentrus* species. In this 3D reconstruction, these antero‐lateral bulges do, however, not appear to be connected to the membranous areas of the auditory bullae. This is because of the shrinking of the swim bladder during specimen fixation and the fact that the tunica interna cannot be seen in μCTscan. (C) The bilateral headphone structures in the anterior part of the swim bladder cover laterally the whole membranous areas of the auditory bullae in *Myripristis* species. DV, dorsal view; LV, lateral view.

The swim bladder of *B. decadactylus* is L‐shaped, with a horizontal part running along the vertebral column and a vertical portion along the vertical haemal spine (Figure 10A). In Holocentrinae, the swim bladder is a simple horizontal tube, extending through the body cavity (Figure 10B). Within this subfamily, the swim bladder of the *Holocentrus* is quite distinctive since the anterior tip lacks the tunica externa, allowing two antero‐lateral bulges of the tunica interna to contact the auditory fenestra (Figure 11B). There is no connection between the swim bladder and the auditory bullae in other Holocentrinae genera (Figure 11A). In Myripristinae, the swim bladder is more complex (Figure 10C). It consists of a main posterior chamber and two antero‐lateral projections joining the auditory fenestrae found in the otic region of the skull (Figure 11C). The anterior projections are linked to the main chamber by a tubular passage whose shape and vertebral position vary across genera. In *Myripristis*, the connection has a narrow constriction at the level of vertebra VI. In *Plectrypops*, it is wider and found under vertebra III. In *Pristilepis* and *Ostichthys*, it lies at the level of vertebra II, whereas there is no apparent constriction in *Corniger* (Figure 10C).

**FIGURE 12:**
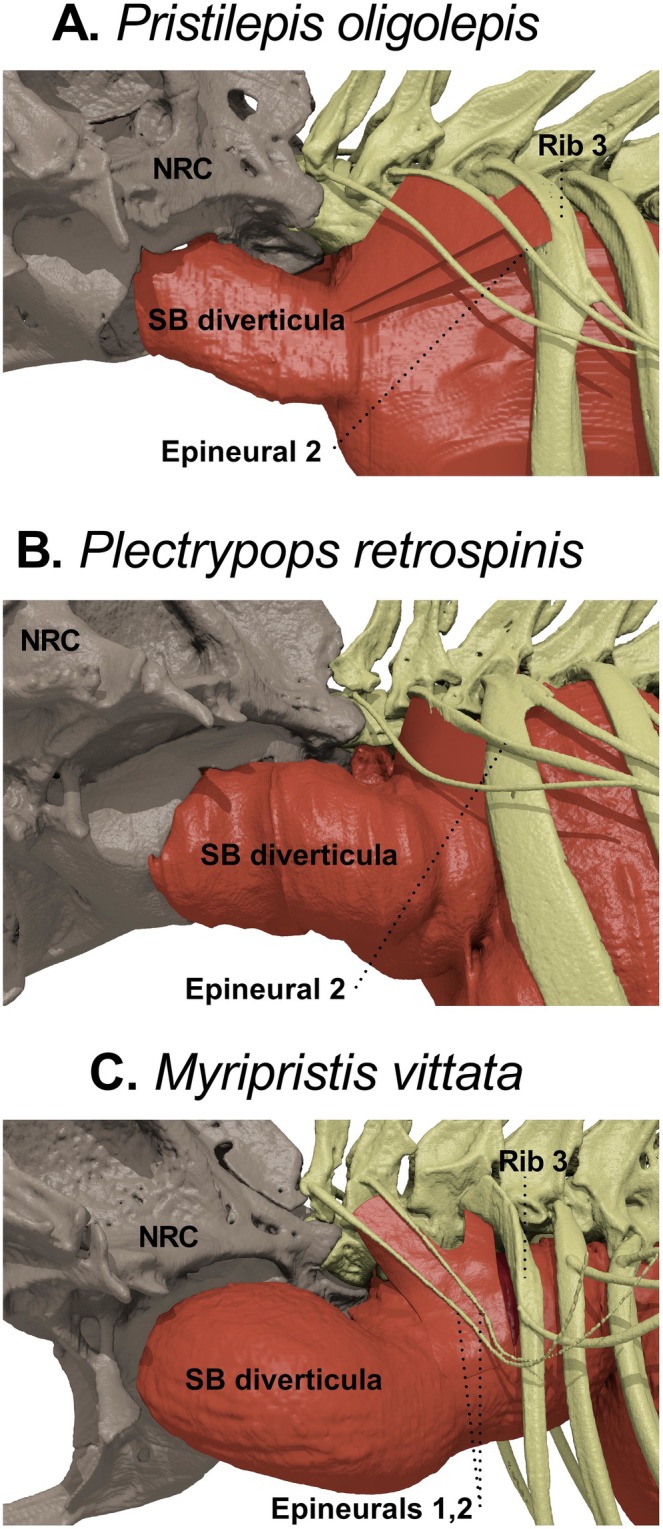
Lateral views of the 3D reconstructions of the different types of insertions of the tunica externa of the swim bladder (SB) on the axial skeleton of representatives of the Myripristinae. (A) In *Pristilepis oligolepis* and *Corniger spinosus* , two bands of tunica externa insert on the 2nd epineural and the 3rd rib. (B) In *Plectrypops retrospinis* and *Ostichthys trachypoma* , a single band of tunica externa inserts onto the 2nd epineural. (C) In *Myripristis vittata* and *Myripristis violacea* , a single band of tunica externa divides into two folds which attach on both the 1st and 2nd epineurals and the 3rd rib. Swim bladder fenestrae are hidden by the tunica externa in this figure. NRC, neurocranium.

In Myripristinae, one or two bands of the tunica externa detach from the swim bladder and insert to the axial skeleton, originating in the formation of the swim bladder fenestra, exclusively made of the tunica interna. In *P. oligolepis* and *C. spinosus* , two bands insert on the 2nd epineural and 3rd rib, respectively (Figure 12A). In *C. spinosus* , the first band additionally inserts on vertebra II. In *P. retrospinis* and *O. trachypoma* , a single band connects to the 2nd epineural (Figure 12B). In *M. vittata* , the band divides into two folds attaching to the ventral head of the 1st and 2nd epineurals and to rib 3 (Figure 12C). In *M. violacea* , a single band attaches to the 1st and 2nd epineurals.

#### 3.2.4 Sonic Muscle Insertions

While the general morphology of the skull, swim bladder, and axial skeleton is conserved within each holocentrid genus, the organization of the sonic muscles, ligaments, and tendinous structures exhibits notable interspecific variation. In all species, sonic muscles originate from the posterior part of the skull, at the level of the pterotic, epiotic, and opisthotic bones that delineate the posterior part of the posttemporal fossa (Figures 4 and 6) and extend caudally, engaging intermuscular structures associated with the first five or six vertebrae.

In Holocentrinae, the muscle typically arises from the caudal edge of the posttemporal fossa and consistently inserts on the 3rd rib, with ligaments extending to ribs 4 and 5. However, structural variations are evident across genera. In *Flammeo*, the muscle inserts dorsally on the 1st and 2nd epineurals and ventrally on rib 3. In *Holocentrus*, the muscle bypasses the 1st epineural and inserts on the 2nd epineural and rib 3, with tendons branching toward ribs 4 and 5. In both *Neoniphon* and *Sargocentron*, the muscle passes beneath the 1st epineural, yet differs in the precise ligamentous configuration. In *Neoniphon*, ligaments form a cascading connection from epineurals to ribs 3 through 5. In *Sargocentron*, insertions on the 2nd epineural and rib 3 are complemented by ligaments extending to ribs 4 and 5, but their orientation and branching differ from *Neoniphon*.

In Myripristinae, the muscle origin varies among genera. In *Corniger* and *Plectrypops*, it resembles that of Holocentrinae. In *Myripristis* and *Pristilepis*, the origin extends more anteriorly over the posttemporal fossa, involving the pterotic and sphenotic bones. This enlarged zone of origin suggests a potentially stronger contraction force. All Myripristinae share a consistent insertion on rib 3 and a serial arrangement of three to four ligaments spanning ribs 3–6. In *Myripristis*, some ligaments also attach to small vertebral apophyses, although their location varies by species. Ligament width and the extent of tendinous coverage differ among taxa.

### 3.3 Acoustic and Morphological Trait Mapping

**FIGURE 13:**
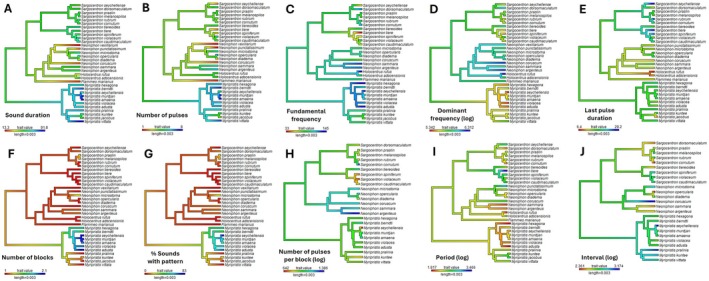
Acoustic traits mapped onto the species tree of Holocentridae using maximum likelihood under the Brownian motion model. (A–J) Acoustic variables of holocentrid sounds investigated by Banse, Bertimes, et al. (2024) and Banse, Minier, et al. (2024). Colors represent trait values observed at the tips or inferred along branches. Trees were pruned to exclude species lacking or inapplicable trait data.

**FIGURE 14:**
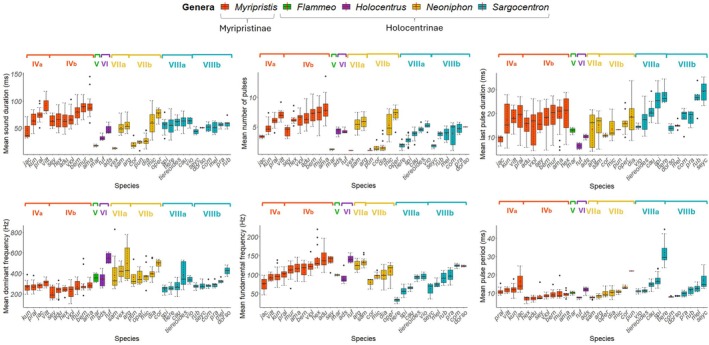
Variation in acoustic parameters across holocentrid species. Boxplots showing the variation of six acoustic variables among the 33 recorded holocentrid species, grouped by subfamily and genus. Within each roman numeral group, species are ordered in ascending value for the corresponding variable. Box boundaries represent the first and third quartiles, whiskers indicate minimum and maximum values, the central lines denote the medians, and red dots indicate the means. Roman numerals correspond to the clades defined in the phylogenetic tree. Species abbreviations: *Ama =* *M. amaena* , *adu =* *M. adusta* , *ads =* *H. adscensionis* , *arg =* *N. argenteus* , *bern =* *M. berndti* , *cau =* *S. caudimaculatum* , *cor = N. coruscum*, *corn =* *S. cornutum* , *dia = N. diadema*, *dorso =* *S. dorsomaculatum* , *hex =* *M. hexagona* , *jac =* *M. jacobus* , *kun =* *M. kuntee* , *mar =* *F. marianus* , *mel =* *S. melanospilos* , *mic =* *N. microstoma* , *mur =* *M. murdjan* , *oper =* *N. opercularis* , *pra =* *S. praslin* , *pral =* *M. pralinia* , *pun = N. punctatissimum*, *ruf =* *H. rufus* , *sam =* *N. sammara* , *rub =* *S. rubrum* , *sey =* *M. seychellensis* , *seyc =* *S. seychellense* , *spi =* *S. spiniferum* , *tiere =* *S. tiere* , *tiereoides = S. tiereoides*, *vex = N. vexillarium*, *vio =* *S. violaceum* , *viol =* *M. violacea* , *vitt =* *M. vittata* .

Acoustic traits mapped onto the phylogeny (33 species; Figure 13) reveal substantial overlap across holocentrid clades, particularly among closely related species. No clear segregation is observed among lineages, as none consistently exhibits extreme values for the defined parameters. These patterns are consistent with those previously identified through comparative analyses (Figure 14) (Banse, Bertimes, et al. 2024). Nonetheless, some broad trends can be identified. For example, *Myripristis* generally produce longer sounds with more pulses than the other genera within the Holocentrinae (Figure 14A,B). Within this genus, a subset of species is further characterized by sounds organized into blocks, as well as shorter pulse periods, higher fundamental frequency, and lower dominant frequency (Figure 14C,D,F,I), compared with its sister clade.

**FIGURE 15:**
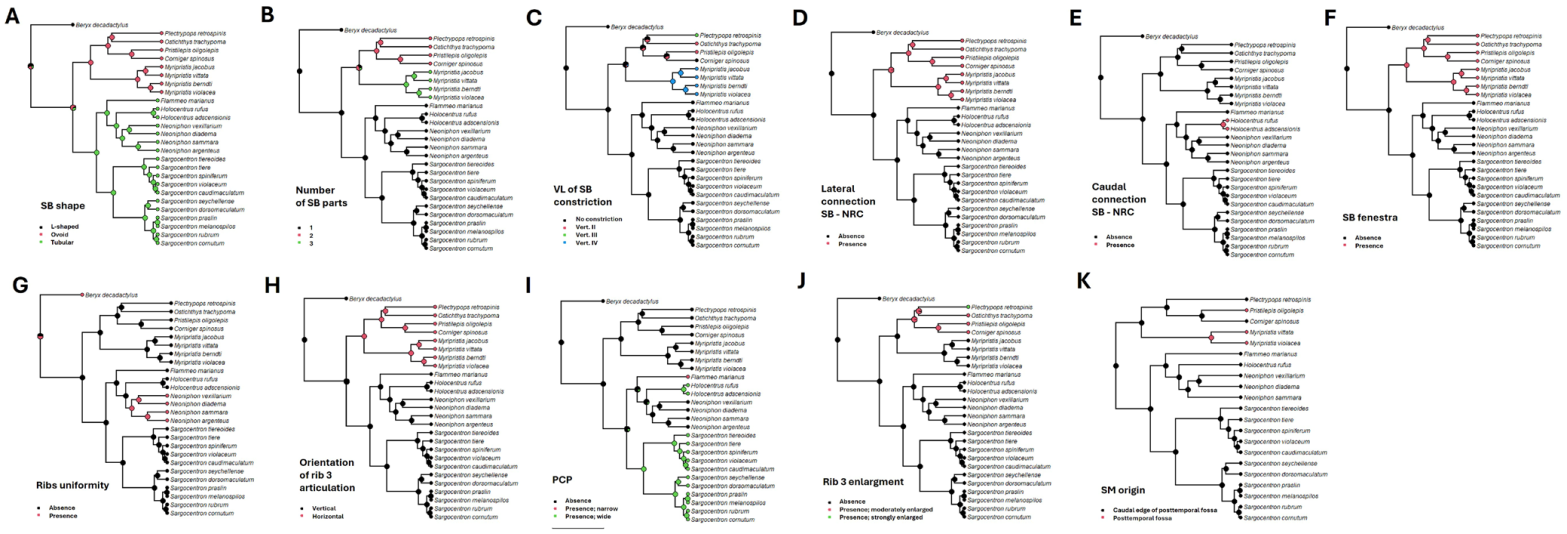
Morphological discrete traits mapped onto the species tree of Holocentridae using stochastic character mapping under the equal‐rates model. Colors at the tips represent observed states, whereas pie charts at internal nodes indicate posterior probabilities of ancestral states. Trees were pruned to exclude species for which trait data were unavailable or inapplicable. (A) Swim bladder (SB) shape; (B) number of SB parts; (C) vertebral level (VL) of SB constriction; (D) lateral connection between the SB and neurocranium (NRC); (E) caudal connection between the SB and NRC; (F) SB fenestra; (G) rib uniformity; (H) orientation of the rib 3 articulation; (I) proximal costal plateau (PCP); (J) rib 3 enlargement; (K) sonic muscle (SM) origin.

To further explore the morphological variation underlying these acoustic patterns, discrete morphological characters were mapped onto the phylogeny (27 species; Figure 15). Relative to the inferred ancestral condition, characterized by a simple‐chambered swim bladder lacking a fenestra and a horizontally oriented 3rd rib (Figure 15B,F,H), the Myripristinae exhibit an increased number of swim bladder chambers, the appearance of a fenestra, and a vertically oriented, thicker 3rd rib. These trends are further accentuated within this lineage, with a higher number of chambers in *Myripristis* (Figure 15B) and a particularly enlarged 3rd rib in *Plectrypops* (Figure 15J). In contrast, Holocentrinae retain several ancestral features but show independent acquisitions, including the development of relatively well‐developed PCP on ribs 3–5 in *Holocentrus* and *Sargocentron*, which are narrower in *Flammeo*, whereas *Neoniphon* lacks these structures (Figure 15I). *Neoniphon* is further distinguished from the other holocentrid genera by the uniformity of its ribs (Figure 15G). Finally, the shape of the swim bladder differs between the Myripristinae and the Holocentrinae, and both diverge from the outgroup *B. decadactylus* (Figure 15A).

## 4 Discussion

Reproductive and advertisement calls are often assumed to convey most species‐specific acoustic information in fishes. However, this assumption has rarely been tested rigorously, and existing studies generally focus on a limited number of closely related taxa. Moreover, even when species‐specific acoustic traits are described, there is currently no evidence that they act as prezygotic barriers or play a decisive role in species recognition. Consequently, the contribution of reproductive sounds to species isolation in fishes remains unclear. In contrast, several comparative studies show that disturbance or agonistic sounds can also display strong species specificity, both in our dataset (Banse, Bertimes, et al. 2024) and in other teleost groups such as pomacentrids (Colleye et al. 2011) or serrasalmids (Mélotte et al. 2019). While aggressive calls differ among species, they nonetheless retain consistent species‐level acoustic signatures. Our objective is not to investigate the evolution of reproductive or social behaviors, but to assess how temporal acoustic patterns produced by a shared sonic mechanism diversify among species. In this context, disturbance calls are particularly suitable: they are produced by all holocentrids under standardized conditions, rely on the same neuromuscular apparatus as social calls, and thus provide a robust and comparable framework for examining acoustic diversification across the family.

In bioacoustics, various constraints such as habitat adaptation, species recognition, or mutations are considered drivers of diversification (Horvatić et al. 2021; Velásquez et al. 2013; Percy et al. 2006). In this study, we examined whether acoustic traits shared among species (Banse, Bertimes, et al. 2024) correspond to synapomorphies, as it is the case for morphological features of the sound‐producing mechanism. By comparing morphology, acoustic features, and phylogeny, we wanted to determine whether acoustic diversity in Holocentridae reflects an evolutionary pattern consistent with their phylogenetic history, or whether it results from more localized, lineage‐specific modifications. We also seek to assess whether this acoustic diversity reflects evolutionary divergence within the family and may be associated with lineage diversification.

Our results are consistent with an evolutionary scenario in Holocentridae involving three main steps: (1) the emergence of a sound‐producing structure; (2) structural differentiation between major lineages; and (3) fine‐scale diversification through neurophysiological modulation of a conserved mechanism; highlighting clear patterns of evolutionary divergence associated with the emergence and modification of the sound‐producing system across lineages. However, linking these evolutionary changes to diversification processes remains challenging, as their macroevolutionary consequences are often lineage‐dependent and difficult to infer from phylogenetic patterns alone (Rabosky 2017; Larouche et al. 2020). In this context, our findings should be interpreted as evidence of evolutionary divergence rather than as direct support for a role of acoustic innovations in diversification processes. This stepwise framework provides a basis for interpreting both morphological and acoustic patterns within the family and highlights how structural innovations combined with behavioral flexibility can generate substantial phenotypic diversification across lineages. Importantly, the differences described here, particularly at higher taxonomic levels (e.g., between subfamilies and genera), involve marked reorganizations of the sound‐producing system, suggesting that they reflect evolutionary divergence among lineages. The strong congruence between acoustic traits, morphology and phylogeny further indicates that acoustic communication is tightly linked to the evolutionary history of the group and is not randomly distributed across taxa.

### 4.1 Evolutionary History

Holocentridae form a sister clade to the Berycidae within the Beryciformes (Near and Thacker 2024). Although earliest Paleocene holocentrid fossils have been described (Andrews et al. 2023), none preserve structures associated with the sonic apparatus. Consequently, the timing and early evolution of sound‐production mechanisms cannot be assessed from the fossil record and must therefore be inferred exclusively from extant lineages. Interestingly, *B. decadactylus* shares key traits with Holocentridae, such as a posttemporal fossa, epineurals, and rib articulations involved in sonic mechanisms, indicating similar abilities. The recurrent involvement of muscles acting on vertebrae‐associated elements in holocentrids, coupled with their occipital origin Holocentrinae (Gainer et al. 1965; Carlson and Bass 2000) and their common innervation by the occipital nerve indicating derivation from somites associated with the posterior part of the skull (Onuki and Somiya 2007) suggest that sonic mechanisms likely evolved through an exaptation process, whereby muscles initially involved in body movement acquired a new role in sound production (Parmentier et al. 2017). The divergence between Myripristinae and Holocentrinae represents a major mechanical differentiation: Myripristinae evolved a bladder directly connected to the skull through anterior projections and ligaments, whereas Holocentrinae retained a simpler, free bladder uncoupled from the cranium. This shift coincides with the earliest phylogenetic split and the first broad differences in sound patterning. Beyond this divergence, acoustic diversification occurred without obvious additional changes in the gross morphology of the sound‐producing apparatus: closely related species share the same structural design yet produce distinct temporal sound patterns, suggesting that signal diversification primarily reflects differences in motor activation and neuromuscular control rather than the evolution of novel structures.

### 4.2 Integrative Evolutionary Scenario

The development of a sound producing apparatus represents the emergence of a new morphotype associated with a new adaptive zone (Hunter 1998; Mayr 1989). Minor structural modifications can subsequently allow for functional specialization, potentially facilitating lineage divergence. In many taxa, these modifications can simply result from one or several changes of an ancestral body plan (Zelditch and Fink 1996). This concept is clearly demonstrated in Holocentridae. Our analyses support the existence of two major lineages (the Myripristinae and the Holocentrinae) that have each evolved separately from a shared ancestral sound‐producing system composed of the swim bladder, the rostral part of the axial skeleton and the bilateral sonic muscles. This division into two lineages could be associated with the auditory apparatus. Modifications in the sound‐producing structures likely stem from subsequent reorganizations involving the anterior positioning of the swim bladder and its contact with the auditory fenestra, which in turn constrained the evolution of associated muscles and ribs. Simultaneously, both taxa have accumulated acoustic divergence in their calls: *Myripristis* produce sounds that are generally longer, composed of more pulses with higher fundamental frequencies and shorter periods compared to Holocentrinae (Banse, Bertimes, et al. 2024). However, establishing a direct link between sound type and sound‐producing structures remains challenging, as the primary differences lie in temporal parameters likely influenced by neuromuscular adaptations rather than by the morphology of the mechanism itself.

Within these two groups, some morphological modifications of the basic theme occur, but they do not fully explain acoustic diversification, which mainly concerns temporal traits and may therefore also reflect differences in the use and motor control of the conserved mechanism (Figure 15).

#### 4.2.1 Focus on the Myripristinae

All Myripristinae possess a swim bladder composed of a main chamber and two antero‐lateral diverticula that insert on the auditory fenestra at the posterior end of the neurocranium. They are also characterized by an increase in the thickness of the 3rd ribs, that additionally articulate vertically on the neural arch and by muscles inserting directly or via a tendon on both the 1st and 2nd epineurals. Although the exact function is not currently understood, all myripristine species also possess bands of tunica externa of the swim bladder that attach to the axial skeleton and/or to the epineurals. Because it is only present in the Myripristinae, this distinctive morphological characteristic suggests that this system has evolved from a common ancestor. Using these lines of evidence and knowing *Myripristis* and *Ostichthys* species are able to make sounds, the species belonging to the genera *Corniger*, *Pristilepis*, and *Plectrypops* are most probably all able to communicate acoustically.

Within the Myripristinae, the morphology of the sound‐producing mechanism of *Myripristis* species gives the impression of a more specialized system, both in terms of sound production and reception. Species of the other genera within the subfamily indeed exhibit less derived features, such as broad ligamentous bands between ribs, sonic muscles that are not always clearly discernible, and auditory fenestrae lacking the basioccipital projection observed in *Myripristis* (Ladich and Popper 2004; Ladich 2014), *Myripristis* appears to combine specializations in both systems. This suggests a possible case where evolutionary coupling between sound production and auditory sensitivity has contributed to the ecological and species diversification within the genus.

#### 4.2.2 Focus on the Holocentrinae

The morphology of the sound‐producing mechanism in the Holocentrinae is fully consistent with the monophyly of this subfamily. However, over the past centuries, the phenotypic similarity among the Holocentrinae has complicated their taxonomic classification (Dornburg et al. 2012).

The genus *Neoniphon* was, for instance, described based on the presence of an elongate body, a large anal spine, and the last spine of the dorsal fin being in close association with the soft dorsal fin (Castelnau 1875). A few years later (Jordan and Evermann 1898), described *F. marianus* based on the presence of a long projecting lower jaw. It was, however, later recognized as a synonym of *Neoniphon* (Randall and Heemstra 1985, 1986). A decade ago, the genus *Flammeo* was resurrected based on the most recent phylogeny of Holocentridae (Dornburg et al. 2012). Because *Neoniphon* and *Sargocentron* were strongly supported as paraphyletic, the authors proposed a revision of the taxonomy of nine additional species classified as *Neoniphon* to *Sargocentron* to match the distinction of the Holocentrinae into four clades in the phylogenies, including the clade formed by *F. marianus* alone and the clade composed of *Holocentrus* species (Dornburg et al. 2012). The reassignment concerned *N. suborbitalis*, *N. vexillarium*, *N. inaequalis* , *N. coruscum*, *N. punctatissimum*, *N. diadema*, *N. microstoma* , *N. ittodai*, and *N. xantherythrum*. In our study, *S. hormion* is classified among the *Neoniphon*. This most likely simply reflects the absence of this species in the phylogenetic trees of Dornburg et al. (2012).

Our phylogenetic data reinforce the revised taxonomy of several holocentrine species (Dornburg et al. 2012). Moreover, it is also supported by both acoustical and morphological data. Sounds indeed enable the discrimination of the four genera within the Holocentrinae (Figure 1) (Banse, Bertimes, et al. 2024). Similarly, a few distinctive morphological features support the distinction of the Holocentrinae into four genera (Figure 1) and, consequently, the reassignment of *S. vexillarium* and *S. diadema* among the *Neoniphon* and, of *N. marianus* as *F. marianus* . *Neoniphon vexillarium* and *N. diadema* possess uniform ribs, similarly to *Neoniphon* species investigated, whereas *Flammeo*, *Holocentrus* and *Sargocentron* species are characterized by the presence of a proximal costal plateau (PCP) on ribs 3–5. This also supports the revised taxonomy of *F. marianus* as such because this species possesses a PCP on ribs 3–5. These PCPs are however more pronounced in *Holocentrus* and *Sargocentron*. Since this character is shared by all Holocentrinae genera but *Neoniphon*, we can hypothesize, under the law of parsimony, that the presence of a PCP on the first ribs consist in the ancestral form. Besides, *F. marianus* also distinguishes from all other genera by the presence of two small ventral processes below vertebra II. *Holocentrus* species are distinguished by the intimate connection between their swim bladder and their auditory fenestra and by their ventral processes at the base of the 2nd epineurals where a bundle of sonic fibers attaches. *Sargocentron* species possess an elongated and forked ventral bony protuberance marking the rostral end of the swim bladder. These characteristics support the distinction of four genera within the Holocentrinae but should be confirmed by the study of additional species, such as for instance *S. hormion* classified among the *Neoniphon* or *N. aurolineatus* , recovered as a sister‐species of *F. marianus* , in our phylogeny (Figure 1).

Our phylogeny suggests that *Sargocentron* first diverged from the remaining Holocentrinae before evolving into two main clades (VIIIa, VIIIb; Figure 1). From a common ancestor then would have evolved four lineages, represented by *Flammeo*, *Holocentrus*, and both *Neoniphon* species of clades VIIa and VIIb. *Holocentrus* spp. are only found in the Atlantic Ocean (Briggs 1974) and could have evolved following the closure of the Isthmus of Panama that divided the East Pacific and western Atlantic oceans approximately 3.1 Ma (Coates and Obando 1996). This hypothesis is fully supported by the estimation of the age of the genus (Dornburg et al. 2015).

Despite minor anatomical differences, species within the same clade mostly produce similar sounds (Banse, Bertimes, et al. 2024), suggesting that interspecific acoustic variation is not strictly linked to morphology. Subtle changes in muscle size or attachment may instead affect the system's energetic efficiency.

### 4.3 Congruence Between Data at Species Level

In holocentrids, there is a congruence between variation in the sound‐producing mechanism morphology and DNA‐based relationships among taxa at subfamily and genus levels. Additionally, the sounds of the two subfamilies and the five recorded genera have specific characteristics that distinguish them from other groups (Banse, Bertimes, et al. 2024). At subgenus and species levels, both congruences and incongruences between phylogenetic and acoustical data levels can be observed.

For instance, the genus *Myripristis* can be divided into two distinct groups of species according to their acoustic features, and these two groups precisely match the two clades of *Myripristis* (IVa and IVb) in the phylogenetic tree (Figure 1). Species belonging to clade IVb produce sounds composed of several blocks of pulses. The use of such an acoustic feature (i.e., short disyllabic or polysyllabic elements) is a capability that, in certain insects, indicates an unusual mechanism of song recognition (Grzywacz et al. 2017). Clade IVa lacks these particular temporal patterns. Within *Sargocentron*, the sounds of species belonging to clade VIIIa ( *S. tiere* , *S. tiereoides*, *S. caudimaculatum* and *S. spiniferum* ) are also more similar than they are from the sounds of their sister clade ( *S. cornutum* , *S. rubrum* , *S. praslin* and *S. melanospilos* ) (Banse, Bertimes, et al. 2024; Figure 1). While the mapping of acoustic and morphological traits onto the phylogeny reveals patterns consistent with phylogenetic structuring, closely related species can nonetheless produce markedly different sounds. For instance, *N. diadema* is closely related to *N. opercularis* (Figure 1) but the sounds of *N. diadema* consist in short sounds < 46 ms made of 1–4 pulses while those of *N. opercularis* last 58 ms at minimum and are composed of 4–9 pulses (Banse, Bertimes, et al. 2024). The evolution of the acoustic signals in phylogenetically close species should exhibit a series of common traits due to their shared ancestor. However, if these species are sympatric, they must also evolve distinct signals to avoid communication interference that could lead to unwanted mating. Conversely, phylogenetically distant species, which also differ by other characteristics besides acoustic signals, might show convergence in the sounds they produce.

In Gobiidae (Horvatić et al. 2021), reported a correlation between acoustic divergence and genetic distance in soniferous European *Gobius* lineage suggesting that certain acoustic features could carry a phylogenetic signal. As suggested in Gobiidae, both the morphology of the sound‐producing mechanism and the acoustic signals seem to carry a phylogenetic signal in Holocentridae, at least to a certain degree. Variation in the degree of association between phylogeny and both morphological and acoustical features was indeed reported in fishes (Amorim and Neves 2007; Malavasi et al. 2008; Horvatić et al. 2019; Amorim et al. 2004). The few discordances observed between molecular, morphological, and acoustical data in holocentrids suggest that other constraints shape the acoustical signals. Among these constraints, selective pressures such as adaptation to the environment (Muñoz et al. 2020; Bradbury and Vehrencamp 1998; Lugli et al. 2003; Lugli 2010; Amorim et al. 2018), divergence in morphology (Mélotte et al. 2019; Malavasi et al. 2008; Muñoz et al. 2020; Parmentier et al. 2022) or selection for species recognition (Spanier 1979; Parmentier et al. 2009) act to amplify the divergence in signals across species (Horvatić et al. 2021). The existence of some discordances between molecular and acoustic data in holocentrids could therefore reflect an additional influence of these constraints on the acoustical signals.

### 4.4 Integration Within a Global Framework

Congruence between acoustic, morphological, and genetic data has been shown in Gobiidae (Malavasi et al. 2008) and Batrachoididae (Rice and Bass 2009). In Holocentridae, this congruence depends on the taxonomic level considered. Although acoustic patterns correspond largely to taxa delimitation at supra‐specific levels (Banse, Bertimes, et al. 2024), variations in the morphology of their sound‐producing mechanism do not fully account for differences in acoustical features. This may partly result from focusing primarily on temporal and spectral parameters, whereas other features such as the pulse shape or sustained sound production capacity are often overlooked. Pulse shape, for example, reflects the combined action of muscles and rib–swim bladder movements.

In fish sound production, both temporal and spectral features do not rely only on anatomy but also on the interaction between neural firing, muscle contraction abilities and morphology of the sound‐producing apparatus. Variations in sound characteristics between taxa could be the result of both fine variations in the sonic mechanism and a differential active control by the vocal motor system of the sonic muscles (Bass and McKibben 2003). Our results mainly show that, during evolution, behavioral modifications (e.g., sound production) that rely on the neurology could appear before modifications of the phenotype as observed in birds (Uy et al. 2018), since different species sharing a similar mechanism can produce distinct signals. Although we did not directly measure neural activity, several temporal sound features analyzed here (notably pulse period) are themselves direct outputs of the firing rhythm imposed by the vocal motor system (Bass et al. 1994). In fishes with fast sonic muscles, each pulse results from a discrete neural command driving a single contraction, meaning that the temporal structure of sounds provides an indirect but functionally meaningful window into neuromuscular control. Differences in temporal patterns among species can therefore arise from divergence in the timing properties of the motor system even when the sonic morphology remains conserved. Similarly, comparative work on swim bladder‐based sonic mechanisms (Fine and Parmentier 2015, 2022) shows that morphological differences alone cannot fully account for interspecific variation in temporal patterns. Although our study does not include new neurophysiological data, the temporal structure of holocentrid disturbance calls can be interpreted as an indirect read‐out of vocal motor control acting on a conserved sound‐producing apparatus. Several studies on other taxa support this assumption that modifications in the vocal signature do not systematically require modifications at the level of the sound‐producing mechanism anatomy. In comparison of 14 *Amphiprion* (clownfish, Pomacentridae) species, the sonic mechanism is highly conservative (Colleye et al. 2011). In this group, sounds are not subject to sexual selection since they are produced during agonistic interactions and interspecific variation in acoustical signals between clownfish species seem rather to be a by‐product resulting from size dimorphism (Colleye et al. 2011). In the Carapini, species of the same genus can produce different kinds of sound using the same basic mechanism but other differences in the acoustic signals produced by species from sister‐genera could be related to fine modifications of the sonic mechanism (Parmentier et al. 2008). In Serrasalmidae, increasing complexity of the sound‐producing mechanism across supra‐generic taxa enabled the production of more complex acoustic signals. However, species within the same genus can also produce different kinds of sounds, which are not strictly species‐specific (Mélotte et al. 2019).

In coral reef fishes, sound production often co‐occurs with distinctive color and motor patterns, which together enhance species recognition (Myrberg and Riggio 1985). In cichlids, females preferentially choose conspecific males and, when this is impossible, select males with the most similar color pattern, indicating that body coloration can act as a reproductive barrier (Couldridge and Alexander 2002). Comparable differences in external morphology and coloration occur in Holocentridae and likely contribute to social interactions. Holocentrinae typically display elongated bodies and conspicuous color patterns such as horizontal stripes, whereas Myripristinae exhibit deeper bodies and generally uniform reddish pigmentation.

Because long wavelengths (yellow to red) are rapidly absorbed in water, reddish fishes become dark and cryptic at depth or at night (Johnsen 2014; Warrant and Adam Locket 2004). This provides an adaptive camouflage for Myripristinae, many of which forage in dim‐light environments or inhabit deeper waters (Greenfield 2002; Greenfield et al. 2017). In contrast, Holocentrinae often exhibit brighter and more contrasting fin or body markings, increasing their visual distinctiveness. These visual signals, combined with acoustic cues, likely facilitate heterospecific discrimination and may reduce selective pressure for highly species‐specific calls in Holocentrinae.

During daylight, visual cues probably dominate species recognition in Holocentridae, allowing acoustic signals to remain relatively unspecific. However, at night—the primary period of activity for many holocentrids—visual information is limited, and reliance on sound should increase. Nocturnal vocal species are known to produce more stereotyped calls than diurnal species (Ruppé et al. 2015; Bertucci et al. 2020), which may explain the presence of more distinctive acoustic codes in some Myripristinae. Thus, ecological differences in visibility and camouflage likely shape the degree of constraint on acoustic communication across the family.

### 4.5 Conceptual Inference

This hierarchical mode of evolution illustrates how modular organization promotes functional evolvability. Once a structural module achieves functional stability, diversification can proceed through changes in neural control and behavioral use rather than through further morphological innovation. Such a framework extends classic models of adaptive radiation by explicitly incorporating a neurofunctional dimension, where behavioral diversification precedes and potentially guides morphological evolution. Comparable decoupling between structure and use occurs in other communication systems. In frogs, similar laryngeal morphologies produce highly divergent call patterns through neural modulation; in songbirds, changes in motor‐pattern generation have diversified song structure without major syringeal modifications; and in mammals, identical skeletal and muscular arrangements underlie a wide range of vocal behaviors.

The holocentrid case therefore exemplifies a general evolutionary rule: once a functional architecture becomes efficient and stable, further diversification is driven by behavioral innovation rather than repeated structural change. While the possibility that signal variation can arise from changes in motor control rather than anatomy has been hinted at in several taxa, it has never been demonstrated at a macroevolutionary scale. In toadfishes, Bass and colleagues (Bass and Baker 1990; Rice and Bass 2009) showed that distinct call patterns can result from differences in motor‐neuron firing within a conserved sonic mechanism. Similar dissociations between structure and acoustic output have been described in other vertebrates, including frogs (Gerhardt et al. 2003) and songbirds (Goller and Suthers 1996; Elemans et al. 2015). However, these studies focused on physiological or species‐level differences rather than on evolutionary transitions across lineages.

## 5 Conclusion

This study provides one of the first evolutionary reconstructions of sound‐production mechanisms in fishes, using Holocentridae as a model. It shows how, from a common ancestral system, structural innovations combined with neurophysiological modulation have shaped acoustic communication across multiple taxonomic levels. Morphological differentiation primarily characterizes higher‐level taxa, whereas functional and behavioral diversification emerges at finer scales. The congruence between morphological, acoustic, and phylogenetic patterns illustrates how subtle modifications in the use of conserved structures can drive diversification and may be associated with lineage differentiation.

These results demonstrate the value of incorporating acoustic traits into evolutionary analyses to better understand the dynamics of species diversification in fishes. Together, they outline a general model for the evolution of communication systems: morphological innovation establishes the structural conditions for sound production, and subsequent neural and behavioral diversification explores the range of possibilities within this framework. This hierarchical process shows that evolutionary novelty often arises through the flexible redeployment of conserved architectures, a principle that may apply broadly across vertebrates.

## Author Contributions

**Marine Banse:** data curation (equal), formal analysis (lead), investigation (lead), methodology (equal), visualization (equal), writing – original draft (equal). **Maarten Van Steenberge:** formal analysis (equal), methodology (equal), resources (equal), validation (equal), writing – review and editing (equal). **Gontran Sonet:** formal analysis (equal), methodology (equal), resources (equal), validation (equal), writing – review and editing (equal). **Denisa Magda:** formal analysis (equal). **Yves Corbisier de Harbonnier de Cobreville:** software (equal), visualization (equal). **David Lecchini:** funding acquisition (equal), investigation (equal), resources (equal), writing – review and editing (equal). **Terry J. Donaldson:** funding acquisition (equal), resources (equal), writing – review and editing (equal). **Eric Parmentier:** conceptualization (equal), formal analysis (equal), funding acquisition (equal), investigation (equal), methodology (equal), resources (equal), software (equal), supervision (equal), validation (equal), visualization (equal), writing – original draft (equal).

## Funding

This work was supported by Agence Nationale de la Recherche, ANR‐19‐CE14‐0010‐SENSO, ANR‐19‐CE34‐0006‐Manini, ANR‐23‐SSRP‐0020‐01, Fonds De La Recherche Scientifique ‐ FNRS, T.0192.20, National Science Foundation, OIA #1946352.

## Conflicts of Interest

The authors declare no conflicts of interest.

## Supporting information


**Appendix S1:** Summary of the DNA sequences collected for each specimen included in the phylogenetic analysis. Information on the morphology of the sonic system and associated acoustic data for each species is also indicated. (*n*) refers to the total number of species for which data is available in the related column, and (*N*) refers to the total number of species investigated in the related column. Sonic system: F = full 3D reconstruction, *P* = partial 3D reconstruction (i.e., no reconstruction of the sonic muscles and ligaments); sound: *R* = recorded. For some specimens in the “Specimens” column, an identification code provide the specimen number and the collection locality (e.g., 1PF stands for individual #1 from French Polynesia): FJ = Fiji, GU = Guam, MAD = Madagascar, PF = French Polynesia, PH = Philippines, REU = Réunion, SAI = Saipan, SEY = Seychelles, SL = Sri Lanka, TAI = Taiwan, TON = Tonga, *Unk* = Unknown.
**Appendix S2:** Summary of the models of DNA substitution and substitution rates applied to each partition in the different genes for ML and BI analyses, respectively. P1, 2, 3 = position 1, 2, 3 in the codon.
**Appendix S3:** Summary of the 17 holocentrid species selected (and the outgroup *Beryx decadactylus* ) for the study of the sonic system. “SM ‐ L rendering” indicates whether the sonic muscles (SM) and ligaments (L) were added on the 3D reconstructions of the sonic system: GD = Guadeloupe, PF = French Polynesia, * refers to specimens from the collection of the Laboratory of Functional and Evolutionary Morphology (ULiège).
**Appendix S4:1** Phylogenetic relationships of the studied holocentrids, constructed using maximum likelihood (ML) and based on concatenated nuclear and mitochondrial gene fragments. The numbers at nodes represent the bootstrap supports (%).
**Appendix S4:2** Phylogenetic relationships of the studied holocentrids, constructed using Bayesian inference (BI) and based on concatenated nuclear and mitochondrial gene fragments. The numbers at nodes represent the bootstrap supports (%).
**Appendix S4:3** Phylogenetic relationships of the studied holocentrids, constructed using maximum likelihood (ML) and based on concatenated nuclear gene fragments. The numbers at nodes represent the bootstrap supports (%).
**Appendix S4:4** Phylogenetic relationships of the studied holocentrids, constructed using Bayesian inference (BI) and based on concatenated nuclear gene fragments. The numbers at nodes represent the Bayesian posterior support.
**Appendix S4:5** Phylogenetic relationships of the studied holocentrids, constructed using maximum likelihood (ML) and based on *COI* mitochondrial DNA. The numbers at nodes represent the bootstrap supports (%).
**Appendix S4:6** Phylogenetic relationships of the studied holocentrids, constructed using Bayesian inference (BI) and based on *COI* mitochondrial DNA. The numbers at nodes represent the Bayesian posterior support.
**Appendix S4:7** Phylogenetic relationships of the studied holocentrids, constructed using maximum likelihood (ML) and based on the nuclear *ENC1* gene fragment. The numbers at nodes represent the bootstrap supports (%).
**Appendix S4:8** Phylogenetic relationships of the studied holocentrids, constructed using Bayesian inference (BI) and based on the nuclear *ENC1* gene fragment. The numbers at nodes represent the Bayesian posterior support.
**Appendix S4:9** Phylogenetic relationships of the studied holocentrids, constructed using maximum likelihood (ML) and based on the nuclear *Glyt* gene fragment. The numbers at nodes represent the bootstrap supports (%).
**Appendix S4:10** Phylogenetic relationships of the studied holocentrids, constructed using Bayesian inference (BI) and based on the nuclear *Glyt* gene fragment. The numbers at nodes represent the Bayesian posterior support.
**Appendix S4:11** Phylogenetic relationships of the studied holocentrids, constructed using maximum likelihood (ML) and based on the nuclear *myh6* gene fragment. The numbers at nodes represent the bootstrap supports (%).
**Appendix S4:12** Phylogenetic relationships of the studied holocentrids, constructed using Bayesian inference (BI) and based on the nuclear *myh6* gene fragment. The numbers at nodes represent the Bayesian posterior support.
**Appendix S4:13** Phylogenetic relationships of the studied holocentrids, constructed using maximum likelihood (ML) and based on the nuclear *Ptr* gene fragment. The numbers at nodes represent the bootstrap supports (%).
**Appendix S4:14** Phylogenetic relationships of the studied holocentrids, constructed using Bayesian inference (BI) and based on the nuclear *Ptr* gene fragment. The numbers at nodes represent the Bayesian posterior support.
**Appendix S4:15** Phylogenetic relationships of the studied holocentrids, constructed using maximum likelihood (ML) and based on the nuclear *sreb* gene fragment. The numbers at nodes represent the bootstrap supports (%).
**Appendix S4:16** Phylogenetic relationships of the studied holocentrids, constructed using Bayesian inference (BI) and based on the nuclear *sreb* gene fragment. The numbers at nodes represent the Bayesian posterior support.
**Appendix S4:17** Species tree of Holocentridae based on six DNA markers (*COI*, *ENC1*, *Glyt*, *myh6*, *Ptr* and *sreb*) and estimated using multispecies coalescent Bayesian inference.
**Appendix S5:** Definitions of the anatomical structures described in this study on Holocentridae. Definitions were established based on the references listed in the corresponding column.
**Appendix S6:** Summary of the position and shape of the auditory fenestra of the auditory bullae on the neurocranium for the 17 investigated species.
**Appendix S7:** Comparative summary of the rostral axial skeleton in the berycid *Beryx decadactylus* and the investigated holocentrid species within each subfamily (SF). The table indicates the vertebral level (VL) of the first parapophyses (PP‐1) and of the swim bladder (SB) constriction, and the articulation sites (AS) of ribs 3 to 6. For ribs 3 and 4, proximal morphology (PM) was characterized from sagittal sections of the body as: wide, when enlarged in the horizontal plane; tall, when enlarged in the vertical plane; and concave, when lits surface exhibited a concavity. PCP indicates the presence of a horizontal proximal costal plateau overlying the rib. The symbols representating these proximal morphologies are illustrated for rib 3. H = Holocentrinae, M = Myripristinae, NA = neural arch, PP‐1/PP‐2 = parapophyses 1 or 2, VB‐3/VB‐4/VB‐5/VB‐6 = vertebral body of vertebra 3, 4, 5 or 6.

## Data Availability

All relevant data are within the article and its Supporting Information.
