# Supplementary material for: Echoes of Evolution in Holocentridae: Harmony Between Phylogeny, Morphology and Acoustics
Source: Ecol Evol. 2026 Aug 19;16(8):e74152. doi: 10.1002/ece3.74152 (PMC13487471; doi:10.1002/ece3.74152)
Supplement: Supplementary file 1 [file ECE3-16-e74152-s001.docx]

Appendix S1. Summary of the DNA sequences collected for each specimen included in the phylogenetic analysis. Information on the morphology of the sonic system and associated acoustic data for each species is also indicated. (n) refers to the total number of species for which data is available in the related column, and (N) refers to the total number of species investigated in the related column. Sonic system: F = full 3D reconstruction, P = partial 3D reconstruction (i.e., no reconstruction of the sonic muscles and ligaments); sound: R = recorded. For some specimens in the “Specimens” column, an identification code provide the specimen number and the collection locality (e.g., 1PF stands for individual #1 from French Polynesia): FJ = Fiji, GU = Guam, MAD = Madagascar, PF = French Polynesia, PH = Philippines, REU = Réunion, SAI = Saipan, SEY = Seychelles, SL = Sri Lanka, TAI = Taiwan, TON = Tonga, *Unk* = Unknown.

|  | Phylogenetics | | | | | | | Morphology | Acoustics |
| --- | --- | --- | --- | --- | --- | --- | --- | --- | --- |
| Specimens  (n = 154) | **Tissue code** | ***COI*  (n = 154)** | ***ENC1*  (n = 146)** | ***Glyt***  **(n = 151)** | ***myh6*  (n = 150)** | ***Ptr*  (n = 146)** | ***sreb***  **(n = 146)** | **Sonic system**  **(N = 18)** | **Sound**  **(N = 34)** |
| *Beryx decadactylus* (outgroup) | YFTC 13625 | AB679219 | JX390750 | JX390790 | JX390829 | JX390869 | JX390936 | P |  |
| *Corniger spinosus* | FDA / USNM 434777 / USNM 426766 | CFSAN18511 | PX093306 | PX093185 | PX097384 | PX093411 | PX093523 | F |  |
| *Flammeo marianus* | YFTC 21765 | JX390734 | JX390768 | JX390808 | JX390847 | - | JX390954 | F | R |
| *Holocentrus adscensionis* | KU114 / YFTC 21876 | JX390748 | JX390751 | JX390791 | JX390830 | JX390870 | JX390937 | P | R |
| *Holocentrus rufus* | YFTC 16195 | JQ840116 | JX390752 | JX390792 | JX390831 | JX390871 | JX390938 | F | R |
| *Myripristis adusta 1PF* | D5_AB49103601 | PX000385 | PX093307 | PX093186 | PX097385 | PX093412 | PX093524 |  | R |
| *Myripristis adusta 1SEY* | A5_AB48766493 | PX000386 | - | PX093187 | PX097386 | PX093413 | PX093525 |  |  |
| *Myripristis adusta 2PF* | E5_AB49103602 | PX000387 | PX093308 | PX093188 | PX097387 | PX093414 | PX093526 |  |  |
| *Myripristis adusta 4GU* | C1_AB48766515 | PX000388 | PX093309 | PX093189 | PX097388 | PX093415 | PX093527 |  |  |
| *Myripristis adusta 6GU* | D1_AB48766514 | PX000389 | - | PX093190 | PX097389 | PX093416 | PX093528 |  |  |
| *Myripristis adusta 6SEY* | B5_AB48766492 | PX000390 | - | PX093191 | PX097390 | PX093417 | PX093529 |  |  |
| *Myripristis adusta PH / SAI* | KU 5598 | FJ583669 | JX390753 | JX390793 | JX390833 | JX390872 | JX390939 |  |  |
| *Myripristis amaena 1PH* | D8_AB48766472 | PX000391 | PX093310 | PX093192 | PX097391 | PX093418 | PX093530 |  | R |
| *Myripristis amaena 4PF* | D4_AB49103595 | PX000392 | PX093311 | PX093193 | PX097392 | PX093419 | PX093531 |  |  |
| *Myripristis amaena 5PF* | E4_AB49103596 | PX000393 | PX093312 | PX093194 | PX097393 | PX093420 | PX093532 |  |  |
| *Myripristis amaena 6GU* | A1_AB48766517 | PX000394 | PX093313 | PX093195 | PX097394 | PX093421 | PX093533 |  |  |
| *Myripristis amaena 6PF* | F4_AB49103597 | PX000395 | PX093314 | PX093196 | PX097395 | PX093422 | PX093534 |  |  |
| *Myripristis amaena 8GU* | B1_AB48766516 | PX000396 | PX093315 | PX093197 | PX097396 | PX093423 | PX093535 |  |  |
| *Myripristis amaena PF / SAI* | KU 5565 | HM034218 | JX390754 | JX390794 | JX390834 | JX390873 | JX390940 |  |  |

| *Myripristis berndti 1GU* | A7_AB49103610 | PX000397 | PX093316 | PX093198 | PX097397 | PX093424 | PX093536 | P | R |
| --- | --- | --- | --- | --- | --- | --- | --- | --- | --- |
| *Myripristis berndti 1PF* | A3_AB49103586 | PX000398 | PX093317 | PX093199 | PX097398 | PX093425 | PX093537 |  |  |
| *Myripristis berndti 1PH* | E6_AB48766483 | PX000399 | PX093318 | PX093200 | PX097399 | PX093426 | PX093538 |  |  |
| *Myripristis berndti 1SEY* | A3_AB48766505 | PX000400 | - | PX093201 | PX097400 | PX093427 | PX093539 |  |  |
| *Myripristis berndti 2GU* | B7_AB49103611 | PX000401 | PX093319 | PX093202 | PX097401 | PX093428 | PX093540 |  |  |
| *Myripristis berndti 2PF* | B3_AB49103587 | PX000402 | PX093320 | PX093203 | PX097402 | PX093429 | PX093541 |  |  |
| *Myripristis berndti 3PF* | C3_AB49103588 | PX000403 | PX093321 | PX093204 | PX097403 | PX093430 | PX093542 |  |  |
| *Myripristis berndti 3PH* | F6_AB48766482 | PX000404 | - | PX093205 | PX097404 | PX093431 | PX093543 |  |  |
| *Myripristis berndti 4GU* | C7_AB49103612 | PX000405 | PX093322 | PX093206 | PX097405 | PX093432 | PX093544 |  |  |
| *Myripristis berndti 4PH* | A7_AB48766481 | PX000406 | PX093323 | PX093207 | PX097406 | PX093433 | PX093545 |  |  |
| *Myripristis berndti PF / Unk* | KU 5043 | HM034161 | JX390755 | JX390795 | JX390835 | JX390874 | JX390941 |  |  |
| *Myripristis botche* | YFTC 22141 | JX390744 | JX390756 | JX390796 | JX390836 | JX390875 | JX390942 |  |  |
| *Myripristis chryseres* | ASIZP0911002 | JX390746 | JX390757 | JX390797 | JX390837 | JX390876 | JX390943 |  |  |
| *Myripristis hexagona 1SEY* | n/a | PX000407 | PX093324 | PX093208 | PX097407 | PX093434 | PX093546 |  | R |
| *Myripristis hexagona 3SEY* | n/a | PX000408 | PX09335 | PX093209 | PX097408 | PX093435 | PX093547 |  |  |
| *Myripristis hexagona 5PH* | n/a | PX000409 | PX093326 | PX093210 | PX097409 | PX093436 | PX093548 |  |  |
| *Myripristis hexagona 6PH* | n/a | PX000410 | PX093327 | PX093211 | PX097410 | PX093437 | PX093549 |  |  |
| *Myripristis hexagona 9PH* | n/a | PX000411 | PX093328 | PX093212 | - | PX093438 | - |  |  |
| *Myripristis hexagona FJ* | KU 4597 | JX390749 | JX390758 | JX390798 | JX390838 | JX390877 | JX390944 |  |  |
| *Myripristis jacobus* | YFTC 21825 | JQ842253 | JX390759 | JX390799 | JX390839 | JX390878 | JX390945 | P | R |
| *Myripristis kochiensis* | FRLM 56669 | PX000412 | - | PX093213 | PX097411 | PX093439 | - |  |  |
| *Myripristis kuntee 1PF* | D3_AB49103589 | PX000413 | PX093329 | PX093214 | PX097412 | PX093440 | PX093550 |  | R |
| *Myripristis kuntee 2GU* | D7_AB49103613 | PX000414 | PX093330 | PX093215 | PX097413 | PX093441 | PX093551 |  |  |
| *Myripristis kuntee 2SEY* | B3_AB48766504 | PX000415 | PX093331 | PX093216 | PX097414 | PX093442 | PX093552 |  |  |
| *Myripristis kuntee 3GU* | E7_AB49103614 | PX000416 | PX093332 | PX093217 | PX097415 | PX093443 | PX093553 |  |  |
| *Myripristis kuntee 3PF* | E3_AB49103590 | PX000417 | PX093333 | PX093218 | PX097416 | PX093444 | PX093554 |  |  |
| *Myripristis kuntee 3PH* | B7_AB48766480 | PX000418 | PX093334 | PX093219 | PX097417 | PX093445 | PX093555 |  |  |

| *Myripristis kuntee 4GU* | F7_AB49103615 | PX000419 | PX093335 | PX093220 | PX097418 | PX093446 | PX093556 |  |  |
| --- | --- | --- | --- | --- | --- | --- | --- | --- | --- |
| *Myripristis kuntee 4PH* | C7_AB48766479 | PX000420 | PX093336 | PX093221 | PX097419 | PX093447 | PX093557 |  |  |
| *Myripristis kuntee 5PF* | F3_AB49103591 | PX000421 | PX093337 | PX093222 | PX097420 | PX093448 | PX093558 |  |  |
| *Myripristis kuntee 5SEY* | C3_AB48766503 | PX000422 | PX093338 | PX093223 | PX097421 | PX093449 | PX093559 |  |  |
| *Myripristis kuntee 6SEY* | D3_AB48766502 | PX000423 | PX093339 | PX093224 | PX097422 | PX093450 | PX093560 |  |  |
| *Myripristis kuntee PF / Unk* | YFTC 16277 | HM034232 | JX390760 | JX390800 | JX390840 | JX390879 | JX390946 |  |  |
| *Myripristis leiognathus* | YFTC 22200 | JX390743 | JX390761 | JX390801 | JX390841 | JX390880 | JX390947 |  |  |
| *Myripristis murdjan 1GU* | D8_AB49103619 | PX000424 | PX093340 | PX093225 | PX097423 | PX093451 | PX093561 |  | R |
| *Myripristis murdjan 1SEY* | A4_AB48766499 | PX000425 | PX093341 | PX093226 | PX097424 | PX093452 | PX093562 |  |  |
| *Myripristis murdjan 2GU* | E8_AB49103620 | PX000426 | PX093342 | PX093227 | PX097425 | PX093453 | PX093563 |  |  |
| *Myripristis murdjan 2PH* | A8_AB48766475 | PX000427 | PX093343 | PX093228 | PX097426 | PX093454 | PX093564 |  |  |
| *Myripristis murdjan 3PH* | B8_AB48766474 | PX000428 | PX093344 | PX093229 | PX097427 | PX093455 | PX093565 |  |  |
| *Myripristis murdjan 3SEY* | B4_AB48766498 | PX000429 | PX093345 | PX093230 | PX097428 | PX093456 | PX093566 |  |  |
| *Myripristis murdjan 4GU* | F8_AB49103621 | PX000430 | PX093346 | PX093231 | PX097429 | PX093457 | PX093567 |  |  |
| *Myripristis murdjan 4PH* | C8_AB48766473 | PX000431 | PX093347 | PX093232 | PX097430 | PX093458 | PX093568 |  |  |
| *Myripristis murdjan 4SEY* | C4_AB48766497 | PX000432 | PX093348 | PX093233 | PX097431 | - | PX093569 |  |  |
| *Myripristis murdjan SL / Unk* | YFTC 12631 | FJ459546 | JX390762 | JX390802 | JX390842 | JX390881 | JX390948 |  |  |
| *Myripristis pralinia 10PH* | D7_AB48766478 | PX000433 | PX093349 | PX093234 | PX097432 | PX093459 | PX093570 |  | R |
| *Myripristis pralinia 11GU* | B8_AB49103617 | PX000434 | PX093350 | PX093235 | PX097433 | PX093460 | PX093571 |  |  |
| *Myripristis pralinia 11PH* | F7_AB48766476 | PX000435 | PX093351 | PX093236 | PX097434 | PX093461 | PX093572 |  |  |
| *Myripristis pralinia 12GU* | C8_AB49103618 | PX000436 | PX093352 | PX093237 | PX097435 | PX093462 | PX093573 |  |  |
| *Myripristis pralinia 13SEY* | n/a | PX000437 | PX093353 | PX093238 | PX097436 | PX093463 | PX093574 |  |  |
| *Myripristis pralinia 14SEY* | E3_AB48766501 | PX000438 | PX093354 | PX093239 | PX097437 | PX093464 | PX093575 |  |  |
| *Myripristis pralinia 15SEY* | F3_AB48766500 | PX000439 | PX093355 | PX093240 | PX097438 | PX093465 | PX093576 |  |  |
| *Myripristis pralinia 1PF* | A4_AB49103592 | PX000440 | PX093356 | PX093241 | PX097439 | PX093466 | PX093577 |  |  |
| *Myripristis pralinia 2PF* | B4_AB49103593 | PX000441 | PX093357 | PX093242 | PX097440 | PX093467 | PX093578 |  |  |
| *Myripristis pralinia 3PF* | C4_AB49103594 | PX000442 | PX093358 | PX093243 | PX097441 | PX093468 | PX093579 |  |  |

| *Myripristis pralinia 5GU* | A8_AB49103616 | PX000443 | PX093359 | PX093244 | PX097442 | PX093469 | PX093580 |  |  |
| --- | --- | --- | --- | --- | --- | --- | --- | --- | --- |
| *Myripristis pralinia 9PH* | E7_AB48766477 | PX000444 | PX093360 | PX093245 | PX097443 | PX093470 | PX093581 |  |  |
| *Myripristis pralinia PF / FJ* | KU4018 | HM034233 | JX390763 | JX390803 | JX390843 | JX390882 | JX390949 |  |  |
| *Myripristis randalli* | KU670 | JX390745 | JX390764 | JX390804 | JX390832 | JX390883 | JX390950 |  |  |
| *Myripristis seychellensis* | n/a | PX000445 | PX093361 | PX093246 | PX097444 | - | PX093582 |  | R |
| *Myripristis tiki* | I.46467.079 | PX000446 | PX093362 | PX093247 | - | - | PX093583 |  |  |
| *Myripristis violacea 1PH* | E8_AB48766471 | PX000447 | PX093363 | PX093248 | PX097445 | PX093471 | PX093584 | F | R |
| *Myripristis violacea 2PF* | A5_AB49103598 | PX000448 | PX093364 | PX093249 | PX097446 | PX093472 | PX093585 |  |  |
| *Myripristis violacea 2PH* | F8_AB48766470 | PX000449 | PX093365 | PX093250 | PX097447 | PX093473 | PX093586 |  |  |
| *Myripristis violacea 2SEY* | D4_AB48766496 | PX000450 | PX093366 | PX093251 | PX097448 | PX093474 | PX093587 |  |  |
| *Myripristis violacea 3PF* | B5_AB49103599 | PX000451 | PX093367 | PX093252 | PX097449 | PX093475 | PX093588 |  |  |
| *Myripristis violacea 3PH* | F8_AB47621280 | PX000452 | PX093368 | PX093253 | PX097450 | PX093476 | PX093589 |  |  |
| *Myripristis violacea 3SEY* | E4_AB48766495 | PX000453 | PX093369 | PX093254 | PX097451 | PX093477 | PX093590 |  |  |
| *Myripristis violacea 4SEY* | F4_AB48766494 | PX000454 | PX093370 | PX093255 | PX097452 | PX093478 | PX093591 |  |  |
| *Myripristis violacea 6PF* | C5_AB49103600 | PX000455 | PX093371 | PX093256 | PX097453 | PX093479 | PX093592 |  |  |
| *Myripristis violacea PF / Unk* | YFTC 12630 | HM034163 | JX390765 | JX390805 | JX390844 | JX390884 | JX390951 |  |  |
| *Myripristis vittata* | KU 4220 | JX390747 | JX390766 | JX390806 | JX390845 | JX390885 | JX390952 | F | R |
| *Neoniphon argenteus* | KU4917 | JX390737 | JX390767 | JX390807 | JX390846 | - | JX390953 | P | R |
| *Neoniphon aurolineatus* | FRLM 40009 | PX000456 | PX093372 | PX093257 | PX097454 | PX093480 | PX093593 |  |  |
| *Neoniphon coruscum* | KU 210 / YFTC 21822 | JQ840674 | JX390777 | JX390815 | JX390855 | JX390892 | JX390962 |  | R |
| *Neoniphon diadema 1PH* | C6_AB48766485 | PX000457 | PX093373 | PX093258 | PX097455 | PX093481 | PX093594 |  | R |
| *Neoniphon diadema 1SEY* | E2_AB48766507 | PX000458 | PX093374 | PX093259 | PX097456 | PX093482 | PX093595 |  |  |
| *Neoniphon diadema 2SEY* | F2_AB48766506 | PX000459 | PX093375 | PX093260 | PX097457 | PX093483 | PX093596 |  |  |
| *Neoniphon diadema 3PF* | D2_AB49103583 | PX000460 | PX093376 | PX093261 | PX097458 | PX093484 | PX093597 | P |  |
| *Neoniphon diadema 3PH* | D6_AB48766484 | PX000461 | PX093377 | PX093262 | PX097459 | PX093485 | PX093598 |  |  |
| *Neoniphon diadema 4PF* | E2_AB49103584 | PX000462 | PX093378 | PX093263 | PX097460 | PX093486 | PX093599 |  |  |
| *Neoniphon diadema 6PF* | F2_AB49103585 | PX000463 | PX093379 | PX093264 | PX097461 | PX093487 | PX093600 |  |  |

| *Neoniphon diadema GU* | F6_AB49103609 | PX000464 | PX093380 | PX093265 | PX097462 | PX093488 | PX093601 |  |  |
| --- | --- | --- | --- | --- | --- | --- | --- | --- | --- |
| *Neoniphon diadema REU / TAI* | ASIZP0910955 | JQ350312 | JX390778 | JX390816 | JX390856 | - | JX390963 |  |  |
| *Neoniphon inaequalis* | YFTC 16279 | JX390738 | JX390779 | JX390817 | JX390857 | JX390893 | JX390964 |  |  |
| *Neoniphon ittodai* | ASIZP0910670 | GU207342 | JX390780 | JX390818 | JX390858 | JX390894 | JX390965 |  |  |
| *Neoniphon microstoma* | KU 5567 | HM034264 | JX390782 | JX390820 | JX390860 | JX390896 | JX390967 |  | R |
| *Neoniphon opercularis* | YFTC 16276 / n/a | JX390740 | JX390769 | JX390809 | JX390848 | PX093489 | JX390955 |  | R |
| *Neoniphon punctassimum* | ASIZP0911452 | JQ350316 | JX390784 | JX390822 | JX390862 | JX390898 | JX390969 |  | R |
| *Neoniphon sammara 1PH* | C5_AB48766491 | PX000465 | PX093381 | PX093266 | PX097463 | PX093490 | PX093602 |  | R |
| *Neoniphon sammara 1SEY* | E1_AB48766513 | PX000466 | PX093382 | PX093267 | PX097464 | PX093491 | PX093603 |  |  |
| *Neoniphon sammara 2PH* | D5_AB48766490 | PX000467 | PX093383 | PX093268 | PX097465 | PX093492 | PX093604 |  |  |
| *Neoniphon sammara 2SEY* | F1_AB48766512 | PX000468 | PX093384 | PX093269 | PX097466 | PX093493 | PX093605 |  |  |
| *Neoniphon sammara 3PF* | B1_AB49103575 | PX000469 | PX093385 | PX093270 | PX097467 | PX093494 | PX093606 | F |  |
| *Neoniphon sammara 4PF* | C1_AB49103576 | PX000470 | PX093386 | PX093271 | PX097468 | PX093495 | PX093607 |  |  |
| *Neoniphon sammara 5PF* | A1_AB49103574 | PX000471 | PX093387 | PX093272 | PX097469 | PX093496 | PX093608 |  |  |
| *Neoniphon sammara GU* | B6_AB49103605 | PX000472 | PX093388 | PX093273 | PX097470 | PX093497 | PX093609 |  |  |
| *Neoniphon sammara REU / FJ* | KU4921 | JQ350142 | JX390770 | JX390810 | JX390849 | JX390887 | JX390956 |  |  |
| *Neoniphon suborbitalis* | KU1155 | JX390739 | JX390787 | JX390826 | JX390866 | JX390901 | JX390973 |  |  |
| *Neoniphon vexillarium* | YFTC 16179 | JQ840676 | JX390789 | JX390828 | JX390868 | - | JX390975 | F | R |
| *Neoniphon xantherythrum* | GenBank | DQ521006 | - | - | - | - | - |  |  |
| *Ostichthys japonicus* | USNM 445307 | PX000473 | PX093389 | - | - | PX093498 | - |  | R |
| *Ostichthys kaianus* | FRLM 46469 | PX000474 | - | PX093274 | PX097471 | PX093499 | PX093610 |  |  |
| *Ostichthys trachypoma* | KU1155 | JX390742 | JX390771 | - | JX390850 | JX390888 | JX390957 | P | R |
| *Plectrypops lima* | FMNH 119175 | JQ350231 | JX390772 | JX390811 | JX390851 | JX390889 | JX390958 |  |  |
| *Plectrypops retrospinis* | KU123 | JX390741 | JX390773 | JX390812 | JX390852 | JX390890 | JX390959 | F |  |
| *Pristilepis oligolepis* | NMNZ P.058460 | PX000475 | PX093390 | PX093275 | PX097472 | PX093500 | - | F |  |
| *Sargocentron caudimaculatum 1PH* | E5_AB48766489 | PX000476 | PX093391 | PX093276 | PX097473 | PX093501 | PX093611 |  | R |
| *Sargocentron caudimaculatum 2PH* | F5_AB48766488 | PX000477 | PX093392 | PX093277 | PX097474 | PX093502 | PX093612 |  |  |
| *Sargocentron caudimaculatum 2SEY* | A2_AB48766511 | PX000478 | PX093393 | PX093278 | PX097475 | PX093503 | PX093613 |  |  |

| *Sargocentron caudimaculatum 3PF* | E1_AB49103578 | PX000479 | PX093394 | PX093279 | PX097476 | PX093504 | PX093614 |  |  |
| --- | --- | --- | --- | --- | --- | --- | --- | --- | --- |
| *Sargocentron caudimaculatum 4GU* | D6_AB49103607 | PX000480 | PX093395 | PX093280 | PX097477 | PX093505 | PX093615 |  |  |
| *Sargocentron caudimaculatum 4PF* | D1_AB49103577 | PX000481 | PX093396 | PX093281 | PX097478 | PX093506 | PX093616 | F |  |
| *Sargocentron caudimaculatum 5PF* | F1_AB49103579 | PX000482 | PX093397 | PX093282 | PX097479 | PX093507 | PX093617 |  |  |
| *Sargocentron caudimaculatum 6GU* | C6_AB49103606 | PX000483 | PX093398 | PX093283 | PX097480 | PX093508 | - |  |  |
| *Sargocentron caudimaculatum 6SEY* | B2_AB48766510 | PX000484 | PX093399 | PX093284 | PX097481 | PX093509 | PX093618 |  |  |
| *Sargocentron caudimaculatum MAD / FJ* | KU 4485 | JQ350308 | JX390774 | JX390813 | JX390853 | JX390891 | JX390960 |  |  |
| *Sargocentron cornutum* | YFTC 13564 / n/a | FJ237589 | JX390776 | JX390814 | JX390854 | PX093510 | JX390961 |  | R |
| *Sargocentron dorsomaculatum* | n/a | PX000485 | PX093400 | PX093285 | PX097482 | PX093511 | - |  | R |
| *Sargocentron hormion* | I.46468.023 | PX000486 | - | PX093286 | PX097483 | PX093512 | PX093619 |  |  |
| *Sargocentron melanospilos* | KU 6987 | HM034260 | JX390781 | JX390819 | JX390859 | JX390895 | JX390966 |  | R |
| *Sargocentron praslin* | YFTC 16282 | JX390735 | JX390783 | JX390821 | JX390861 | JX390897 | JX390968 |  | R |
| *Sargocentron rubrum* | YFTC 22126 | IQ623980 | 1X390785 | 1X390823 | 1X390863 | - | 1X390970 |  | R |
| *Sargocentron seychellense* | KU 6908 | JX390736 | JX390786 | JX390824 | JX390864 | JX390899 | JX390971 | F | R |
| *Sargocentron spiniferum 1SEY* | C2_AB48766509 | PX000487 | PX093401 | PX093287 | PX097484 | PX093513 | PX093620 |  | R |
| *Sargocentron spiniferum 3GU* | E6_AB49103608 | PX000488 | PX093402 | PX093288 | PX097485 | PX093514 | PX093621 |  |  |
| *Sargocentron spiniferum 3PF* | A2_AB49103580 | PX000489 | PX093403 | PX093289 | PX097486 | PX093515 | PX093622 |  |  |
| *Sargocentron spiniferum 3PH* | A6_AB48766487 | PX000490 | PX093404 | PX093290 | PX097487 | PX093516 | PX093623 |  |  |
| *Sargocentron spiniferum 4PF* | B2_AB49103581 | PX000491 | PX093405 | PX093291 | PX097488 | PX093517 | PX093624 |  |  |
| *Sargocentron spiniferum 4PH* | B6_AB48766486 | PX000492 | PX093406 | PX093292 | PX097489 | PX093518 | PX093625 |  |  |
| *Sargocentron spiniferum 5PF* | C2_AB49103582 | PX000493 | PX093407 | PX093293 | PX097490 | PX093519 | PX093626 |  |  |
| *Sargocentron spiniferum MAD / TON* | KU753 | JQ350318 | JX390775 | JX390825 | JX390865 | JX390900 | JX390972 |  |  |
| *Sargocentron spiniferum SEY* | D2_AB48766508 | PX000494 | PX093408 | PX093294 | PX097491 | PX093520 | PX093627 |  |  |
| *Sargocentron tiere* | KU 5702 | JQ350322 | JX390788 | JX390827 | JX390867 | JX390902 | JX390974 |  | R |
| *Sargocentron tiereoides* | F5_AB49103603 | PX000495 | PX093409 | PX093295 | PX097492 | PX093521 | PX093628 |  | R |
| *Sargocentron violaceum* | A6_AB49103604 | PX000496 | PX093410 | PX093296 | PX097493 | PX093522 | - |  | R |

Appendix S2. Summary of the models of DNA substitution and substitution rates applied to each partition in the different genes for ML and BI analyses, respectively. P1, 2, 3 = position 1, 2, 3 in the codon.

|  | mtDNA | Nuclear DNA (nDNA) | | | | |
| --- | --- | --- | --- | --- | --- | --- |
|  | *COI* | *ENC1* | *Glyt* | *myh6* | *Ptr* | *sreb* |
| Base pairs | 651 | 695 | 721 | 534 | 561 | 853 |
| *Models applied to each partition in ML analyses (IQ-TREE)* | | | | | | |
| P1 | TN+F+G4 | TIM2e+I | TPM3u+F+I | TIM3e+I | TNe | F81+F |
| P2 | F81+F | HKY+F | K2P+I | HKY+F+I | TN+F+I | TPM2u+F+G4 |
| P3 | TIM3+F+R3 | TPM2u+F+G4 | K2P+G4 | TNe+G4 | TIM2+F+G4 | JC+I+G4 |
| *Substitution rates applied to each partition in BI (Mr Bayes on Cipres Portal)* | | | | | | |
| P1  (Nst = 6) | gamma | propinv | propinv | propinv | equal | equal |
| P2 (Nst = 6) | equal | equal | equal | propinv | propinv | gamma |
| P3  (Nst = 6) | gamma | gamma | gamma | gamma | gamma | invgamma |

Appendix S3. Summary of the 17 holocentrid species selected (and the outgroup *Beryx decadactylus*) for the study of the sonic system. ‘SM - L rendering’ indicates whether the sonic muscles (SM) and ligaments (L) were added on the 3D reconstructions of the sonic system: GD = Guadeloupe, PF = French Polynesia, * refers to specimens from the collection of the Laboratory of Functional and Evolutionary Morphology (ULiège).

| Species | ID | Sample site | Voxel resolution (µm) | SM - L rendering |
| --- | --- | --- | --- | --- |
| *Corniger spinosus* | UF 238259 | Cat’s Paw Reef (Gulf of Mexico) | 73.8 | X |
| *Plectrypops retrospinis* | UF 133606 | Abaca Islands (Bahamas) | 29.7 | X |
| *Ostichthys trachypoma* | UF 72155 | Marco Island (Florida) | 30 |  |
| *Pristilepis oligolepis* | KPM-NI 006833 | Sagami Bay (Japan) | 43.7 | X |
| *Myripristis berndti* | Mbern_4PF* | Moorea (PF) | 19.7 |  |
| *Myripristis jacobus* | Mjac_1GD* | GD | 34.1 |  |
| *Myripristis violacea* | Mviol_4PF* | Moorea (PF) | 44.2 | X |
| *Myripristis vittata* | Mvitt_2PF* | Moorea (PF) | 43.4 | X |
| *Flammeo marianus* | Fmar_4GD* | GD | 36.7 | X |
| *Holocentrus rufus* | Hruf_1GD* | GD | 43.3 | X |
| *Holocentrus adscensionis* | Hads_1GD* | GD | 31.3 |  |
| *Neoniphon sammara* | Nsam_6PF* | Moorea (PF) | 34.3 | X |
| *Neoniphon argenteus* | Narg_5PF* | Moorea (PF) | 30.2 |  |
| *Neoniphon diadema* | Ndia_5PF* | Moorea (PF) | 28.8 | X |
| *Neoniphon vexillarium* | Nvex_2GD* | GD | 24.7 | X |
| *Sargocentron caudimaculatum* | Scau_6PF* | Moorea (PF) | 34.7 | X |
| *Sargocentron seychellense* | Ssey_6SEY* | Seychelles | 51.6 | X |
| *Outgroup (Beryx decadactylus)* | MNHN:2004-2124 |  | 85.1 |  |

Appendix S4.1 Phylogenetic relationships of the studied holocentrids, constructed using maximum likelihood (ML) and based on concatenated nuclear and mitochondrial gene fragments. The numbers at nodes represent the bootstrap supports (%).

Appendix S4.2 Phylogenetic relationships of the studied holocentrids, constructed using Bayesian inference (BI) and based on concatenated nuclear and mitochondrial gene fragments. The numbers at nodes represent the bootstrap supports (%).

Appendix S4.3 Phylogenetic relationships of the studied holocentrids, constructed using maximum likelihood (ML) and based on concatenated nuclear gene fragments. The numbers at nodes represent the bootstrap supports (%).

Appendix S4.4 Phylogenetic relationships of the studied holocentrids, constructed using Bayesian inference (BI) and based on concatenated nuclear gene fragments. The numbers at nodes represent the Bayesian posterior support.

Appendix S4.5 Phylogenetic relationships of the studied holocentrids, constructed using maximum likelihood (ML) and based on *COI* mitochondrial DNA. The numbers at nodes represent the bootstrap supports (%).

Appendix S4.6 Phylogenetic relationships of the studied holocentrids, constructed using Bayesian inference (BI) and based on *COI* mitochondrial DNA. The numbers at nodes represent the Bayesian posterior support.

Appendix S4.7 Phylogenetic relationships of the studied holocentrids, constructed using maximum likelihood (ML) and based on the nuclear *ENC1* gene fragment. The numbers at nodes represent the bootstrap supports (%).

Appendix S4.8 Phylogenetic relationships of the studied holocentrids, constructed using Bayesian inference (BI) and based on the nuclear *ENC1* gene fragment. The numbers at nodes represent the Bayesian posterior support.

Appendix S4.9 Phylogenetic relationships of the studied holocentrids, constructed using maximum likelihood (ML) and based on the nuclear *Glyt* gene fragment. The numbers at nodes represent the bootstrap supports (%).

Appendix S4.10 Phylogenetic relationships of the studied holocentrids, constructed using Bayesian inference (BI) and based on the nuclear *Glyt* gene fragment. The numbers at nodes represent the Bayesian posterior support.

Appendix S4.11 Phylogenetic relationships of the studied holocentrids, constructed using maximum likelihood (ML) and based on the nuclear *myh6* gene fragment. The numbers at nodes represent the bootstrap supports (%).

Appendix S4.12 Phylogenetic relationships of the studied holocentrids, constructed using Bayesian inference (BI) and based on the nuclear *myh6* gene fragment. The numbers at nodes represent the Bayesian posterior support.

Appendix S4.13 Phylogenetic relationships of the studied holocentrids, constructed using maximum likelihood (ML) and based on the nuclear *Ptr* gene fragment. The numbers at nodes represent the bootstrap supports (%).

Appendix S4.14 Phylogenetic relationships of the studied holocentrids, constructed using Bayesian inference (BI) and based on the nuclear *Ptr* gene fragment. The numbers at nodes represent the Bayesian posterior support.

Appendix S4.15 Phylogenetic relationships of the studied holocentrids, constructed using maximum likelihood (ML) and based on the nuclear *sreb* gene fragment. The numbers at nodes represent the bootstrap supports (%).

Appendix S4.16 Phylogenetic relationships of the studied holocentrids, constructed using Bayesian inference (BI) and based on the nuclear *sreb* gene fragment. The numbers at nodes represent the Bayesian posterior support.

Appendix S4.17 Species tree of Holocentridae based on six DNA markers (*COI*, *ENC1*, *Glyt*, *myh6*, *Ptr* and *sreb*) and estimated using multispecies coalescent Bayesian inference.

**Appendix S5. Definitions of the anatomical structures described in this study on Holocentridae.** Definitions were established based on the references listed in the corresponding column.

| **Name** | **Definition** | **Anatomy** |
| --- | --- | --- |
| Epineural | Epineurals are intermuscular bones associated with the neural arch or neural spine of vertebrae, generally positioned above the horizontal septum within the dorsal musculature, although their position may vary along the vertebral column. | Patterson and Johnson (1995) |
| Epipleural | Intermuscular bones associated with the hypaxial musculature, typically attached to or arising near the ribs and extending into the myosepta. | Patterson and Johnson (1995) |
| Rib | Ribs in teleosts are skeletal elements typically preformed in cartilage, articulating on the parapophyses of abdominal vertebrae and located within the peritoneal membrane. | Patterson and Johnson (1995) |
| Parapohysis | A parapophysis is a ventrolateral process of the vertebra that serves as the site of articulation or attachment for the ribs. | Patterson and Johnson (1995) |
| Tunica externa | Outer connective component of the swim bladder wall composed of multiple tissue types, that provides structural support and mechanical integration. | Parmentier *et al.* (2025) |
| Tunica interna | Inner epithelial component lining the lumen of the swim bladder. | Parmentier *et al.* (2025) |
| Posttemporal fossa | Cranial depression located on the caudal surface of the skull, delineated by the pterotic, epiotic and opisthotic bones, and serving as the site of origin for sonic muscles. | This study |
| Swim bladder fenestra | Localized region of the swim bladder wall lacking the tunica externa, resulting in a structurally reduced area of the bladder envelope. | Parmentier *et al.* (2020) |

**References**

Parmentier E, Marucco FE, Millot M, Raick X, Thiry M (2020). Sound production, hearing sensitivity and in-depth study of the sound-producing muscles in the cowfish (*Lactoria cornuta*). Journal of Anatomy **238**:956–969.

Parmentier E, Eche L, Greeven C, Banse M, Bertucci F, Thiry M, Raick X, Donaldson TJ, Lecchini D (2025). Morphological innovations and evolutionary transitions in boxfish acoustic communication, *Biological Journal of the Linnean Society*, **146**, 2.

Patterson C, Johnson GD (1995) The intermuscular bones and ligaments of teleostean ﬁshes. Smithsonian Contribution to Zoology **559**: 1–85.

Appendix S6. Summary of the position and shape of the auditory fenestra of the auditory bullae on the neurocranium for the 17 investigated species.

| Species | Position | Shape |
| --- | --- | --- |
| *Beryx decadactylus* | Antero-ventral | Triangular |
| *Pristilepis oligolepis* | Postero-lateral | Semi-circular |
| *Corniger spinosus* | Postero-lateral | Semi-circular |
| *Plectrypops retrospinis* | Postero-lateral | Semi-circular |
| *Ostichthys trachypoma* | Postero-lateral | Elliptical |
| *Myripristis vittata* | Lateral | Ear-shaped |
| *Myripristis jacobus* | Lateral | Ear-shaped |
| *Myripristis berndti* | Lateral | Ear-shaped |
| *Myripristis violacea* | Lateral | Ear-shaped |
| *Flammeo marianus* | Lateral | Curvilinear triangular |
| *Holocentrus rufus* | Caudal | Circular |
| *Holocentrus adscensionis* | Caudal | Circular |
| *Neoniphon vexillarium* | Lateral | Curvilinear triangular |
| *Neoniphon sammara* | Lateral | Curvilinear triangular |
| *Neoniphon diadema* | Lateral | Curvilinear triangular |
| *Sargocentron caudimaculatum* | Lateral | Curvilinear triangular |
| *Sargocentron seychellense* | Lateral | Curvilinear triangular |

Appendix S7. Comparative summary of the rostral axial skeleton in the berycid *Beryx decadactylus* and the investigated holocentrid species within each subfamily (SF). The table indicates the vertebral level (VL) of the first parapophyses (PP-1) and of the swim bladder (SB) constriction, and the articulation sites (AS) of ribs 3 to 6. For ribs 3 and 4, proximal morphology (PM) was characterized from sagittal sections of the body as: wide, when enlarged in the horizontal plane; tall, when enlarged in the vertical plane; and concave, when lits surface exhibited a concavity. PCP indicates the presence of a horizontal proximal costal plateau overlying the rib. The symbols representating these proximal morphologies are illustrated for rib 3. H = Holocentrinae, M = Myripristinae, NA = neural arch, PP-1/PP-2 = parapophyses 1 or 2, VB-3/VB-4/VB-5/VB-6 = vertebral body of vertebra 3, 4, 5 or 6.

| Species | SF | VL  of PP-1 | VL of SB  constriction | AS  of rib 3 | PM of rib 3 | PM of rib 3 (symbol) | AS  of rib 4 | PM of rib 4 | AS  of rib 5 | AS  of rib 6 |
| --- | --- | --- | --- | --- | --- | --- | --- | --- | --- | --- |
| *Beryx decadactylus* | Outgroup | V | - | VB-3 | Tall |  | VB-4 | - | VB-5 | PP-1 |
| *Pristilepis oligolepis* | M | VII | II | NA | Wide |  | NA | Wide | NA | VB-6 |
| *Corniger spinosus* | M | VII | - | NA | Wide |  | NA | Wide | NA | VB-6 |
| *Plectrypops retrospinis* | M | VII | III | NA | Very wide |  | NA | Wide | NA | VB-6 |
| *Ostichthys trachypoma* | M | VII | II | NA | Wide |  | NA | Wide | NA | VB-6 |
| *Myripristis vittata* | M | V | VI | NA | Concave |  | NA | Wide | PP-1 | PP-2 |
| *Myripristis jacobus* | M | V | VI | NA | Concave |  | NA | Tall | PP-1 | PP-2 |
| *Myripristis berndti* | M | V | VI | NA | Concave |  | NA | Tall | PP-1 | PP-2 |
| *Myripristis violacea* | M | VI | VI | NA | Concave |  | NA | Tall | NA | PP-1 |
| *Flammeo marianus**^1^ | H | VI | - | VB-3 | PCP |  | VB-4 | PCP | VB-5 | PP-1 |
| *Holocentrus rufus**^2^ | H | VI | - | VB-3 | PCP |  | VB-4 | PCP | VB-5 | PP-1 |
| *Holocentrus adscensionis**^2^ | H | VI | - | VB-3 | PCP |  | VB-4 | PCP | VB-5 | PP-1 |
| *Neoniphon vexillarium* | H | VI | - | VB-3 | Tall |  | VB-4 | Tall | VB-5 | PP-1 |
| *Neoniphon sammara* | H | VI | - | VB-3 | Tall |  | VB-4 | Tall | VB-5 | PP-1 |
| *Neoniphon diadema* | H | VI | - | VB-3 | Tall |  | VB-4 | Tall | VB-5 | PP-1 |
| *Sargocentron caudimaculatum**^3^ | H | VI | - | VB-3 | PCP |  | VB-4 | PCP | VB-5 | PP-1 |
| *Sargocentron seychellense**^3^ | H | VI | - | VB-3 | PCP |  | VB-4 | PCP | VB-5 | PP-1 |

*^1^ *Flammeo marianus* possess two ventral bugles below vertebra II

*^2^ *Holocentrus* species possess a ventral process at the base of the 2^nd^ epineurals

*^3^ *Sargocentron* species possess a forked ventral process projecting forward below vertebra II
